# Unleashing the power of microalgae: a pioneering path to sustainability and achieving the sustainable development goals

**DOI:** 10.1007/s11356-025-35885-8

**Published:** 2025-02-08

**Authors:** Shimaa Hosny, Mostafa E. Elshobary, Mostafa M. El-Sheekh

**Affiliations:** 1 National Institute of Oceanography and Fisheries (NIOF), Alexandria, Egypt; 2 Botany and Microbiology Department, Faculty of Science, Tanta University, Tanta, 31527 Egypt; 3 Aquaculture Research, Alfred Wegener Institute (AWI) – Helmholtz Centre for Polar and Marine Research, Am Handelshafen, Bremerhaven, 27570 Germany

**Keywords:** Blue carbon, Phycoremediation, Biofuel, Dye-sensitized solar cells, Bioplastic, Therapeutics, Nutritional applications

## Abstract

This study explores the remarkable potential of algae in addressing global sustainability challenges. Microalgae, in particular, emerge as sustainability champions. Their applications span an impressive array of industries and processes, including food and feed production, biofuels, cosmetics, pharmaceuticals, and environmental remediation. This versatility positions algae as key players in achieving over 50% of UN Sustainable Development Goals (SDGs) simultaneously, addressing issues such as climate action, clean water and sanitation, affordable and clean energy, and zero hunger. From sequestering carbon, purifying wastewater, and producing clean energy to combating malnutrition, algae demonstrates unparalleled potential. Their ability to flourish in extreme conditions and their rapid growth rates further enhance their appeal for large-scale cultivation. As research advances, innovative applications continue to emerge, such as algae-based bioplastics and dye-sensitized solar cells, promising novel solutions to pressing global issues. This study illuminates how harnessing the power of algae can drive us towards a more resilient, sustainable world. By leveraging algae’s multifaceted capabilities, we can tackle climate change, resource scarcity, and economic development concurrently. The research highlights the critical role of algae in promoting circular economy principles and achieving a harmonious balance between human needs and environmental preservation, paving the way for a greener, more sustainable future.

## Introduction

The idea of sustainable development was introduced to acknowledge the interconnectedness of social, environmental, and economic systems and ensure that current generations can meet their requirements without threatening the capacity of future generations to do the same (Olabi et al. 2023). The Sustainable Development Goals (SDGs) were established in 2015 by the United Nations (UNs) as a set of 17 objectives by 193 nations to direct international collaboration towards sustainable development to promote a clean and safe environment and a healthy lifestyle for future generations. The SDGs developed on the Millennium Development Goals (MDGs), from 2000 to 2015 (Obaideen et al. 2021; Sayed et al. 2021). To achieve these objectives, international cooperation is essential. Despite the challenges posed by the COVID-19 pandemic, following the global financial crisis, and weak international collaboration, these goals are critical to addressing issues such as an increasing population, poor communication, limited resources, inadequate policies, political decisions, and insufficient finances faced by every nation (Shehata et al. 2022). These goals highlight key objectives that must be achieved. Solving social, political, and economic issues through sustainable development goals thoroughly investigates the main challenges in stopping climate change and creating green and sustainable industries, jobs, and local economies.

**Fig. 1:**
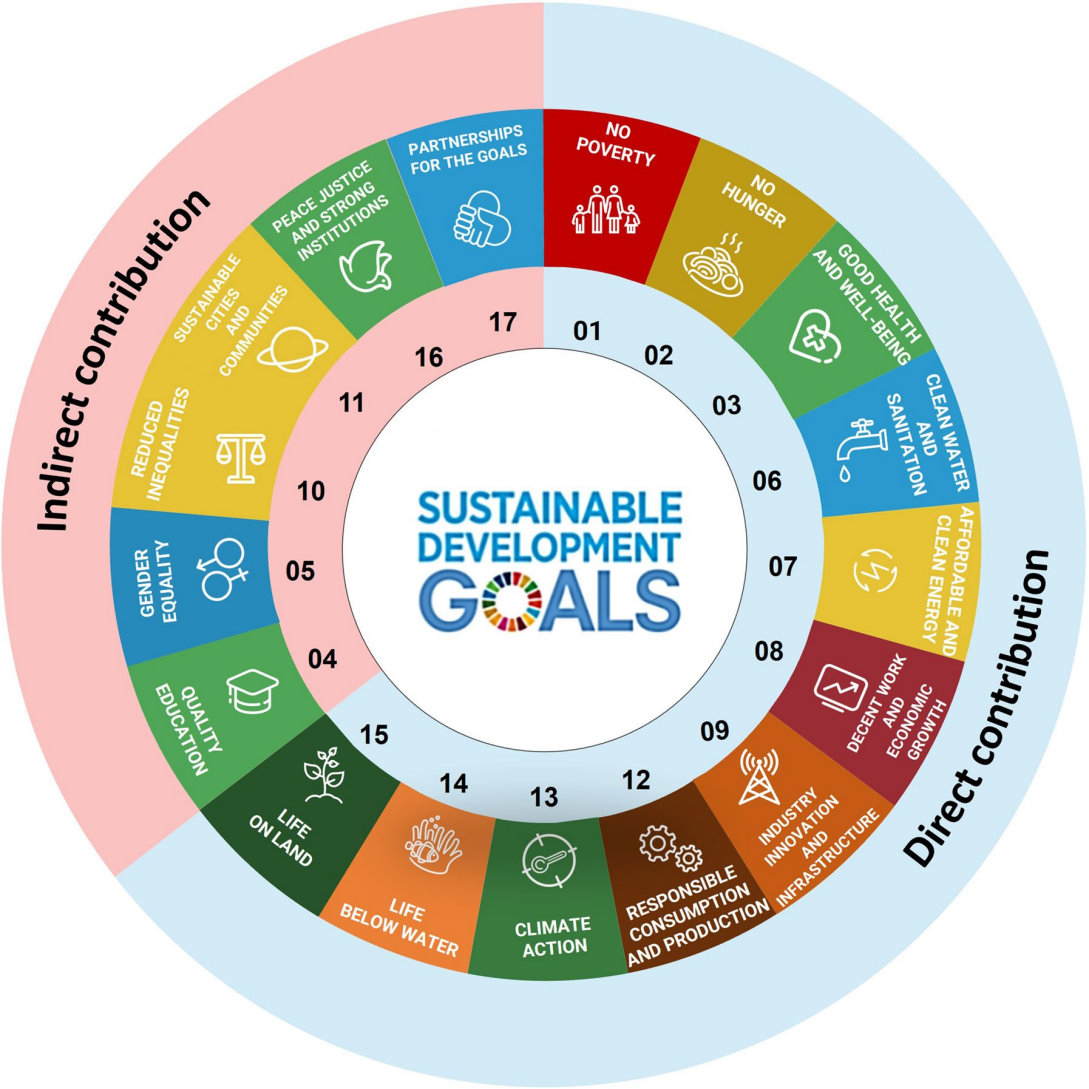
Microalgae contribute both directly and indirectly to the Sustainable Development Goals (SDGs)

It is predicted that the widespread use of algae in the industry could result in achieving over 50% of the Sustainable Development Goals (SDGs), particularly no poverty (SDG 1), zero hunger (SDG 2), good health and well-being (SDG 3), clean water and sanitation (SDG 6), affordable and clean energy (SDG 7), decent work and economic growth (SDG 8), industry, innovation and infrastructure (SDG 9), responsible consumption and production (SDG 12), climate action (SDG 13), life below water (SDG 14), and life on land (SDG 15) (Fig. 1). Additionally, microalgae can contribute indirectly to the remaining SDGs: reduced inequalities (SDG 10), sustainable cities and communities (SDG 11), peace, justice and strong institutions (SDG 16), and partnerships for the goals (SDG 17). These indirect contributions arise from the broader socioeconomic and environmental impacts of algae-based industries, such as fostering innovation, promoting sustainable practices, and creating new educational and partnership opportunities.

Algae are unique, primitive photosynthetic organisms with extraordinary potential that flourish in numerous environments. They are classified mainly into macro- and microalgae that can grow in various habitats with water and sunlight, including soils, ponds, lakes, oceans, rivers, the sea, and tree bark. Algae can withstand different environmental conditions, such as changes in temperature, salinity, pH levels, and light intensity (El Shafay et al. 2021; Kholssi et al. 2023; Osman et al. 2023). The most amazing fact is that microalgae can grow with CO_2_ from atmospheric and industrial flue gas, which is about 10 times higher than terrestrial plants, and they can do so without freshwater or arable land (Feng et al. 2024). As a result, the raw materials needed for microalgal cultivation are commonly available in the atmosphere. Over 80,000 different microalgae species are thought to exist, and about half of them have undergone economic and commercial research. However, the actual number will be substantially greater, depending on how “algae” is defined, with some estimates reaching a million different species (Guiry 2013). Due to their diverse metabolisms, these organisms can produce a wide range of substances with anthropogenic significance, such as functional ingredients, for example, carotenoids produced by *Dunaliella* and *Haematococcus*, the high-quality proteins and carbohydrates found in *Arthrospira* and *Chlorella*. The production of polyunsaturated fatty acids (PUFAs), vitamins (A: retinol, B1: thiamine, B6: pyridoxine, B12: cobalamin, C: ascorbic acid, and E: tocopherol, riboflavin, nicotinic acid, biotin, folic acid, and pantothenate), antioxidants, polysaccharides, and pigments by different algae species is crucial in achieving food safety (Chhandama et al. 2021). Furthermore, algae can also serve as a source of important compounds such as bioenergy (Abomohra and Elshobary 2019; Elshobary et al. 2022). The Aquatic Species Program of the U.S. Energy Department categorized almost 3000 microalgae species based on their potential to produce biofuels through indirect methods.

While there are numerous reviews available that cover different aspects of microalgae farming systems (Singh and Patidar 2018), products derived from microalgae (Sarkar et al. 2020), microalgae’s function in wastewater remediation (Nagarajan et al. 2022), and biofuel production (Ray et al. 2022), few studies have addressed the importance of microalgae in accomplishing the various SDGs. Although Merlo et al. (2021) briefly examined the impact of algae on some of the SDGs, their focus was mainly on sustainable energy (SDG 7), and they did not extensively discuss microalgae’s role in accomplishing the other SDGs. Additionally, the role microalgae play in the circular economy must be clarified. Therefore, this work’s primary purpose is to emphasize algae’s crucial role in achieving sustainable development goals.

## Microalgae’s role in accomplishing the SDGs

**Fig. 2:**
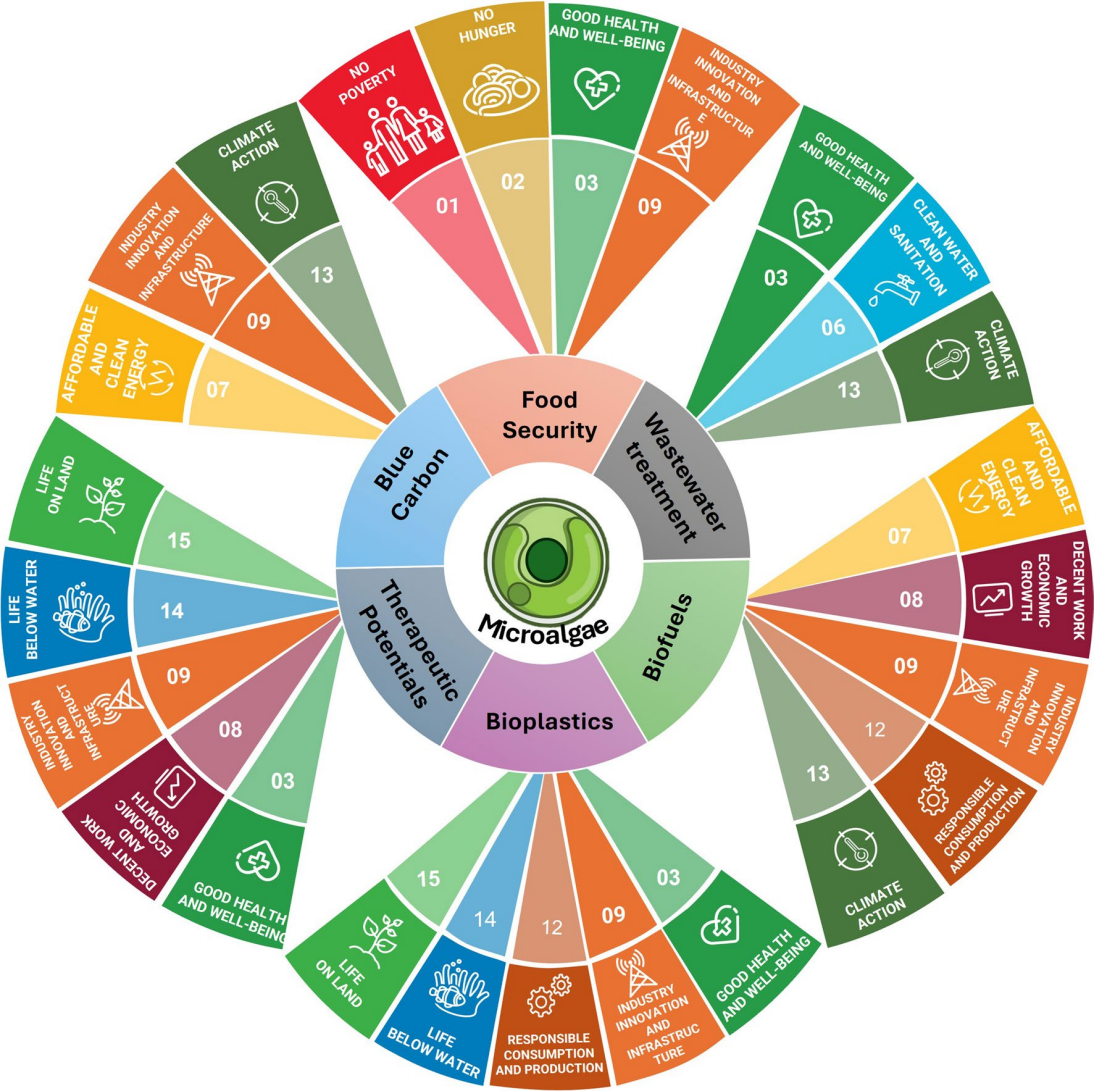
Microalgae significantly contribute directly to more than 50% of the Sustainable Development Goals (SDGs)

Microalgae represent a promising biotechnology that could be crucial for achieving numerous Sustainable Development Goals (Fig. 2). They have several applications, including reducing malnutrition, managing the environment, ensuring food security, providing energy, purifying water, and producing chemicals such as biofertilizers, cosmetics, and healthcare products, in addition to creating jobs and promoting economic growth (Olabi et al. 2023). Moreover, microalgae can significantly impact sustainable development by reducing CO_2_, the main greenhouse gas that causes climate change. Therefore, cultivating microalgae to recycle materials and energy is an important strategy for the circular economy.

It is important to form strong partnerships with the government and business sectors to challenge the existing ways of doing things. Lately, there has been growing awareness from the industrial, commercial, and academic sectors about exploring and creating various valuable products from microalgae, including renewable biofuels, bioactive substances like carotenoids, vitamins, lipids, peptides, lutein, astaxanthin, polyunsaturated fatty acids (PUFA) (Zhou et al. 2022), and cosmetics. These products significantly contribute to different fields and promote the widespread use of microalgae. Additionally, we need to understand better how microalgae can contribute to the circular economy. This study aims to demonstrate how microalgae can help achieve sustainable development goals by producing blue carbon, wastewater treatment, biofuel, dye-sensitized solar cells, bioplastic, medical products, and nutritional products.

### Blue carbon

According to (Singh and Dhar 2019), carbon dioxide acts as a greenhouse gas that absorbs and stores solar radiation in the atmosphere, contributing significantly to global warming and climate change. Climate change is a critical aspect of sustainable development goals that has garnered worldwide attention due to rising sea levels, increased greenhouse gases, longer and more frequent heat waves, loss of Greenland and Antarctic ice sheets, and declining biodiversity (Biermann and Kim 2020). According to Lovelock and Duarte (2019), the term “Blue Carbon” refers to carbon that coastal and oceanic ecosystems, primarily photosynthetic organisms like algae, macroalgae, seagrasses, salt marshes, and mangroves, capture and store. As oceans cover 70% of the earth’s surface, researching the possibilities of blue carbon in the sector is becoming increasingly common (Ricart et al. 2022). However, the effectiveness of Blue carbon as a CO_2_ removal solution remains disputed, with ongoing research showing that these ecosystems, in some cases, remove more carbon per area than land forests (Boyd et al. 2022).

Aquatic photosynthetic organisms, especially phytoplankton, are accountable for half of the world’s carbon sequestration and oxygen production despite comprising only 1% of global plant biomass (Behrenfeld 2014). In light of this, the question arises whether microalgae culture can contribute to blue carbon.

#### Carbon capture by microalgae

Research on carbon capture and sequestration (CCS), a method that encourages the collection and storage of carbon dioxide, has received a lot more funding over the last 20 years. These technologies use solid materials like CaO or liquids like alkyl amines to adsorb CO_2_ from flue gas. After that, the CO_2_ is concentrated by calcination or thermal desorption, which yields a concentrated form suitable for long-term storage. Usually, stable geological formations like deep seas or exhausted oil and gas reservoirs are used to inject concentrated CO_2_, which can be held there for hundreds or even millions of years. However, a difficult risk assessment and close monitoring are necessary due to the geological and physical issues with subterranean or deep ocean CO_2_ storage. In addition to transportation, pressurization, and ongoing monitoring, these storage needs and the cost of CCS increase (IPCC 2018; Realmonte et al. 2019). On the other hand, carbon capture and utilization (CCU) can eliminate the need for carbon storage. Turning CO_2_ from air or point sources into valuable products with lower or no emissions, like chemicals, fuels, carbon fibers, biomass, and building raw materials, is known as carbon capture and utilization (CCU). According to Srinivasan et al. (2021), capturing CO_2_ transforms it from an expense or waste product to an opportunity. Depending on how it is used, this technology could contribute to net-zero and negative emissions. The biological mechanism by which CO_2_ is converted into biomass in microalgae-based CCU, which is ecologically friendly to the environment, is called photosynthesis (Brilman et al. 2013). Microalgae can recycle CO_2_ into bioenergy and are 400 times more effective at fixing CO_2_ than terrestrial plants (Sutherland et al. 2021). This remarkable capacity for carbon sequestration makes algae cultivation an effective tool in the fight against climate change. This approach directly addresses SGD 9 (industry innovation and infrastructure) and SDG 13 (climate action). Moreover, the biomass produced can be used as a renewable feedstock for various industries, further reducing reliance on fossil fuels (Brennan and Owende 2010) that supports SDG 7 (affordable and clean energy) and SDG 8 (decent work and economic growth). It has been estimated that this method can capture about 30,000 t/yr of CO_2_, according to Morales et al. (2018). The biomass produced can replace petroleum and coal by producing chemical compounds, biofuels, bioplastics, and biofertilizers.

Microalgae culture can contribute significantly to carbon sequestration and climate change mitigation, despite the potential release of captured carbon when used as food, feed, or biofuel. Recent research has highlighted several pathways for effective carbon sequestration using microalgae. Temporary carbon storage by microalgae, even if later released, can provide valuable time for implementing other mitigation strategies (Moreira et al. 2023). When used as biofuel, microalgae participates in a short-term carbon cycle, replacing fossil fuels and reducing net emissions (Kumar et al. 2020b). Long-term sequestration can be achieved through methods such as biomass burial in anaerobic environments or conversion to biochar, which can remain stable in soil for centuries (Law et al. 2022). Innovative applications in construction materials and biochemical extraction offer additional avenues for carbon sequestration (Sarwer et al. 2022). Moreover, the high efficiency of microalgae in carbon capture compared to terrestrial plants makes them valuable even if some carbon is eventually released (Sarwer et al. 2022). Coupled systems integrating microalgae cultivation with other carbon capture technologies show promise for enhancing overall sequestration effectiveness (Arun et al. 2020). Some microalgae species can also contribute to ocean alkalinity enhancement, boosting the ocean's capacity to absorb atmospheric CO_2_ (Bach et al. 2021). By maintaining continuous cultivation and exploring these diverse strategies, microalgae systems can play a crucial role in climate change mitigation, balancing carbon capture with practical uses of algal biomass.

Brennan and Owende (2010) reported that *Chlorella* sp. can grow at CO_2_ concentrations between 0.77 and 2.22 g/L/day or 40% (v/v) (Cheah WaiYan et al. 2015). *Oscillatoria,* a freshwater algal, can fix between 70 and 80% of the CO_2_ in the environment at the optimum condition (Anguselvi et al. 2019). *Arthrospira* sp. can enhance its biomass in vertical photobioreactors by up to 18% when carbon dioxide is injected (Ye et al. 2020). Factors such as algal physiology, pond chemistry, temperature, light, mass transfer of CO_2_, and nutrient availability all play a role in determining how effectively microalgae can biocapture CO_2_ (Daneshvar et al. 2022). Flue gas characteristics like CO_2_ concentration, temperature, and toxic substances are also important factors.

Microalgae biomass output cultivated in a 53,000 km^2^ culture area is estimated to absorb 0.5393 Gt CO_2_ each year, with an average annual production of 324.33 million tons of microalgae biomass (Zhao and Su 2020). Microalgae in both open and closed culture modes may produce 280 tons of dry biomass per hectare annually from 513 tons of CO_2_ by using 9% of the light energy they obtain. According to (Khan et al. 2018), published research suggests that 1.0 kg of cultivated microalgae may absorb up to 1.83 kg of CO_2_. Three main ways that microalgae take in inorganic carbon are through extracellular carbonic anhydrase, which changes bicarbonates into CO_2_, the plasma membrane, which lets CO_2_ indirectly, and dissolved inorganic carbon (DIC) pumps on the membrane, which lets bicarbonates indirectly (Shahid et al. 2020a, b). Finally, the challenges in using microalgae-based carbon capture and utilization are cultivation, biomass harvesting, and extracting products (Labeeuw et al. 2021).

#### Air filtration

Along with capturing CO_2_, algae can also filter air from pollutants. According to the World Health Organization (WHO 2018), air pollution significantly contributes to premature deaths worldwide. To address this, the *U.S. Environmental Protection Agency* (EPA) has established guidelines for six air pollutants: lead, sulfur dioxide, carbon dioxide, nitrogen dioxide, ozone in the atmosphere, and particle matter (US-EPA 2014). The increase in air pollution is mainly attributed to primary and secondary air pollutants, such as gaseous pollutants: carbon monoxide (CO), sulfur dioxide (SO_2_), sulfur trioxide (SO_3_), nitrogen monoxide (NO), nitrogen dioxide (NO_2_), nitrous oxide (N_2_O), ammonia (NH_3_), hydrogen sulfide (H_2_S), methane (CH_4_), chlorofluorocarbons (CFCs), and volatile organic compounds (VOCs); liquid pollutants: aerosols; and solid pollutants: particulate matter (PM) (Suresh and Benor 2020). Microalgae can reduce flue gas carbon dioxide levels by half and decrease NO_x_ and SO_x_ levels when batches of flue gas bubbles are injected through photobioreactors (Tripathy et al. 2021). Kao et al. (2014) observed that NO_x_ and SO_x_ in flue gas enhanced the growth of microalgae. Another study found that *Chlorella* sp. KR1 efficiently utilized SOx (50 ppm), improving biomass concentration(Steven 2023). However, some studies have shown that SOx gas pollutants can inhibit microalgae growth. The effect of NO_x_ and SO_x_ on microalgae growth can vary depending on the species, concentration, and other environmental factors. Further research is needed to better understand the interactions between microalgae and these gas pollutants. A recent study examined how CO_2_, SO_2_, NO, and ash from a coal power plant’s flue gas affected the microalga *Chlorella fusca* LEB 111’s ability to fix CO_2_ and grow, as well as how its biomass was characterized. The results showed that adding NO and SO_2_ up to 400 ppm had no significant effect on CO_2_ fixation by the microalga. *Scendesmus dimorphus* is tolerant of elevated NO and CO_2_ concentrations. The suppression of microalgae by flue gas can be addressed by intermittent sparging with pH control and flue gas. Intermittent flue gas sparging produced the highest biomass concentration (3.63 g/L) and CO_2_ biofixation (75.61%). Microalgae can withstand higher CO_2_ levels when they coexist in symbiotic interactions (Jiang et al. 2013). For instance, the White Sea benthic hydroid *D. pumila*'s symbiotic microalga *Desmodesmus* sp. 3Dp86E-1 can withstand up to 20% CO_2_ without experiencing any changes in its ultrastructure or mode of operation. The growth rate of the culture also increased twofold. But for microalgae to grow well at higher CO_2_ levels, the rate at which they take in carbon through photosynthesis and how well they deliver photosynthates for biosynthesis is important (Solovchenko et al. 2015). These abilities make symbiotic microalgae suitable for advanced technologies to mitigate CO_2_. Other microalgae species have also been reported for CO_2_ biomitigation (Table 1). Several factors, including the type of algal strain, culture conditions, media composition, initial pollutant and biomass loads, and the duration of pollutant contact with the algae, influence the efficiency of removing various pollutants. The mode of culture (photoautotrophic, photoheterotrophic, heterotrophic, and mixotrophic) also affects pollutant removal efficiency. Some algae strains isolated from harsh environments have shown the ability to accumulate radioactive and heavy metals (Tripathy et al. 2021).

**Table 1:** Different species of microalgae used for the bioremediation of air pollutants

Algae species	Contaminant Remediated	Air pollutants conc	Reference
*Oscillatoria*	CO_2_	70–80%	(Anguselvi et al. 2019)
*Arthrospira* sp*.*	CO_2_	Sustain 12% CO_2_	(Dębowski et al. 2024)
*Monoraphidium minutam*	CO_2,_ SO_*X*_, and NO_*X*_	13.6%	(Radmann et al. 2011)
*Scenedesmus dimorphus*	CO_2_	75.61%	(López-Sánchez et al. 2022)
*Chlorella vulgaris*	CO_2_	80%	(Sadeghizadeh et al. 2017)
*Chlorocuccum littorale*	CO_2_	50%	(Ota et al. 2009)
*Nannochloropsis oculata*	CO_2_	15%	(Chiu et al. 2009)
*Scenedesmes* sp*.*	NO_X_	300 ppm	(Santiago et al. 2010)
*Scenedesmus dimorphous*	SOx	200 ppm	(Jiang et al. 2013)
*Skeletonema costatum*	CO_2_	5–10%	(Gao et al. 2019; Wu et al. 2022)
*Dunaliella tertiolecta*	NO_X_	300 ppm	(Zhu et al. 2022)

To fully utilize the potential of algae for carbon capture and utilization, several challenges need to be addressed, including the culture system, microalgae, and the extraction of products (Labeeuw et al. 2021).

#### Case study

**Fig. 3:**
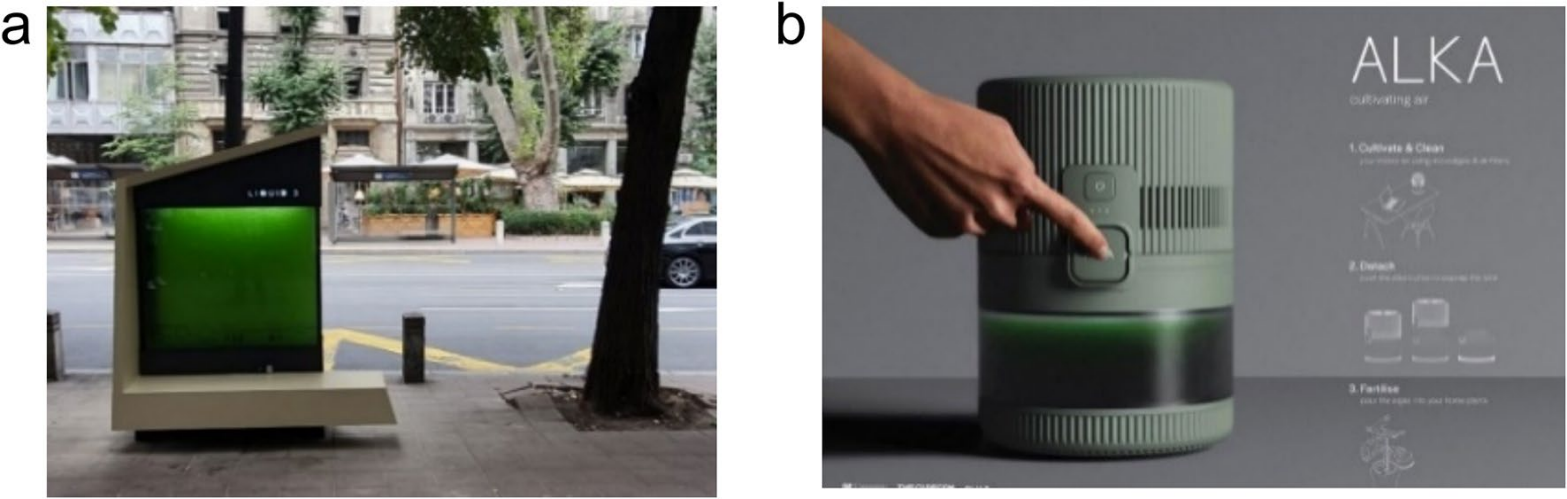
**a** “LIQUID 3” The first algae air purifier in Serbia (United Nations Development Programme 2021). **b **AlKA cultivating air filter (Times of Malta 2021)

The United Nations Development Program and the Ministry of Environmental Protection recognized “LIQUID 3” as one of the top 11 creative and environmentally friendly solutions in the “Climate Smart Urban Development” project. The first urban photo-bioreactor in Serbia, “LIQUID 3,” has been installed on Makedonska Street in Belgrade, in front of the Municipality of Stari Grad (Fig. 3a). It is referred to as the “liquid tree” by the “Institute for Multidisciplinary Research” at the University of Belgrade, where it was created. It is a novel biotechnological solution to address high concentrations of carbon dioxide (CO_2_) and air pollution in urban areas. The photo-bioreactor utilizes algae to absorb CO_2_ and release oxygen through photosynthesis within a 600-l water tank. Additionally, LIQUID 3 is multifunctional as it is designed to function as a bench, with mobile phone chargers and a solar panel to provide lighting at night.

Ben Sammut, a product design engineer who recently graduated from the University of Glasgow and Glasgow School of Art, has created a unique air filter named “Alka FILTER AIR” Fig. 3b. This filter uses *Arthrospira* to capture carbon dioxide. This pollutant has been linked to a loss of concentration. The device “inhales” carbon dioxide and produces oxygen. After two weeks, the device can be transformed into a watering can, and the captured algae can be poured onto plants.

### Wastewater treatment

According to estimates from the United Nations, almost 56% of all freshwater used is dumped as wastewater WW (Urban Waste Water Treatment in Europe ReportUrban Waste Water Treatment in Europe Report 2024). However, despite the investments high-income nations have made in treating WW, relatively little of the treated water is effectively recycled (Hassan et al. 2022). It is vital to look for sustainable procedures that allow wastewater reuse because it is predicted that by 2030 the world will experience a 40% water deficiency (United Nations World Water Development Report 4 2021). Consequently, wastewater remediation is necessary to improve water quality and lessen water deficiency (Karimi-Maleh et al. 2020). It is important to note that when treated water is released into streams, the N_2_, PO_4_, and COD (organic carbon demand) levels must comply with the standard limits set by the European Union Directive 91/271/CEE. Therefore, it is crucial to review the processes used for wastewater treatment.

#### Methods of wastewater remediation

Membrane filtration, electrochemical treatment, ion exchange, precipitation, osmosis, and evaporation are common chemical and physical treatments for wastewater treatment. Nevertheless, these techniques could be more economically viable and environmentally sustainable. A new integrated technology is necessary to save costs and achieve sustainable development goals (Crini and Lichtfouse 2019; Rani et al. 2019). Hence, current research has centered on treating wastewater biologically over chemical treatments. Although using chemical methods to treat wastewater is the fastest, it has harmful impacts on the ecosystem. Therefore, biological wastewater treatment is the most sustainable strategy.

Bioremediation uses microorganisms like bacteria, fungi, and microalgae to eliminate environmental pollutants. Two pathways of bioremediation are bioaccumulation and biosorption. Bioaccumulation is a metabolic process where a live organism respires and utilizes energy to remove pollutants. Biosorption, on the other hand, uses fresh or dried algae to take heavy metal ions out of acidic solutions. This is done through metabolic or physicochemical mechanisms of uptake. It was stated that using microalgae for wastewater treatment can lower the cost of treatment compared to traditional bacterial treatments that require high energy (Mennaa et al. 2015; El-Sheekh et al. 2023b). Microalgae cultivation can fix a high rate of atmospheric CO_2_ and has a high biomass yield greater than terrestrial agriculture and the fastest degradation of pollutants. Therefore, developing microalgae culture may be a viable substitute for the current methods of remediate wastewater. The integration of algae cultivation into wastewater treatment systems contributes to SDG 6 (clean water and sanitation), SDG 3 (good health and well-being), and SDG 13 (climate action) by reducing greenhouse gas emissions. Algae can effectively remove excess nutrients, heavy metals, and other pollutants from wastewater, improving water quality while simultaneously producing valuable biomass (Wang et al. 2024b, c). This dual-purpose approach offers a sustainable solution to water treatment challenges, particularly in regions facing water scarcity.

Urban, industrial, and agricultural wastewater typically contains a high concentration of organic and inorganic substances like NO_3_, C, and PO_4_, which can serve as a nutrient source for microalgae growth (Shahid et al. 2020b). The effects of eutrophication, smog production, and ocean acidification on ecosystems have been significantly mitigated by using microalgae in wastewater treatment (Bussa et al. 2019). Furthermore, growing microalgae in wastewater can be economically and ecologically beneficial due to their high productivity and bioactive compounds, which makes them a promising source of biofuel, organic fertilizers, biostimulants, and high-value compounds. This approach aligns with the circular economy concept and promotes sustainable resource management (El-Shenody et al. 2023).

The European Directive 2008/105/EC requires wastewater treatment plants (WWTPs) to address various emerging pollutants, including biocides, chlorinated solvents, polycyclic aromatic hydrocarbons, pesticides, pharmaceuticals, cosmetics, and personal care products. However, traditional methods for removing organic substances still need to be more effective, consistent, and viable for pharmaceuticals (Santos et al. 2013).

A recent study investigated the sensitivity of several microalgae strains to pharmaceuticals and personal care products (PPCPs) and found that algae play an essential role in wastewater treatments (Wang et al. 2017). The fluoroquinolone antibiotic enrofloxacin was tested on *Tetradesmus obliquus*, *Chlamydomonas Mexicana,* and *Chlorella vulgaris* separately and together. It was found that these species could handle large amounts of drugs and recover quickly (Xiong et al. 2018). Guo et al. (2016) showed that *Chlorella* sp., *Chlamydomonas* sp., and *Mychonastes* sp. microalgae are lipid-rich and very resistant to cephalosporin antibiotics, and they did not show any harmful effects while building up the drug.

#### Example of algal species for WW treatment

To achieve high biomass growth rates and efficient water treatment, choosing strains resistant to toxic components in wastewater is necessary. From wastewater, microalgae can extract heavy metals, nitrogen, phosphate, coliform bacteria, and biochemical and chemical oxygen requirements (Morsi et al. 2023a). Up to 0.063 g of nitrogen and 0.009 g of phosphorus can be removed from an effluent with 1 g of microalgae cell biomass per liter. *Ankistrodesmus falcatus, Botryococcus braunii, Chlamydomonas Mexicana, Chlorella kessleri, Chlorella sorokiniana, Scenedesmus obliquus* (Mennaa et al. 2019),* Chlorella pyrenoidosa* (Xiaogang et al. 2022)* Coleastrum* sp. (Hajinajaf et al. 2021)*, Tetradesmus sp.* (Wang et al. 2024b)*, Scenedesmus obliquus*, *Scenedesmus dimorphus* (Ruangsomboon et al. 2020)* Nannochloropsis* sp. (Emparan et al. 2020),* Chlorococcum* sp. (Morsi et al. 2023b), *Neochloris oleabundans* (Manzoor et al. 2021)*, Oocystis pusilla, Chlorococcus infusionum* (Osman et al. 2023), and* Dunaliella salina* (Takriff et al. 2016) are common species used in WWT.

### Biofuels

The rising concerns over the sustainability of the environment and the security of the world’s energy resources have raised awareness of the importance of sustainable biofuel sources. Among these, biofuels generated through green technology have emerged as a potential solution to these challenges (Ang et al. 2022). Biofuels are liquid, gaseous, and solid fuels derived from biomass through biological processes instead of geological processes (Ganesan et al. 2020; Ismail et al. 2020).

**Fig. 4:**
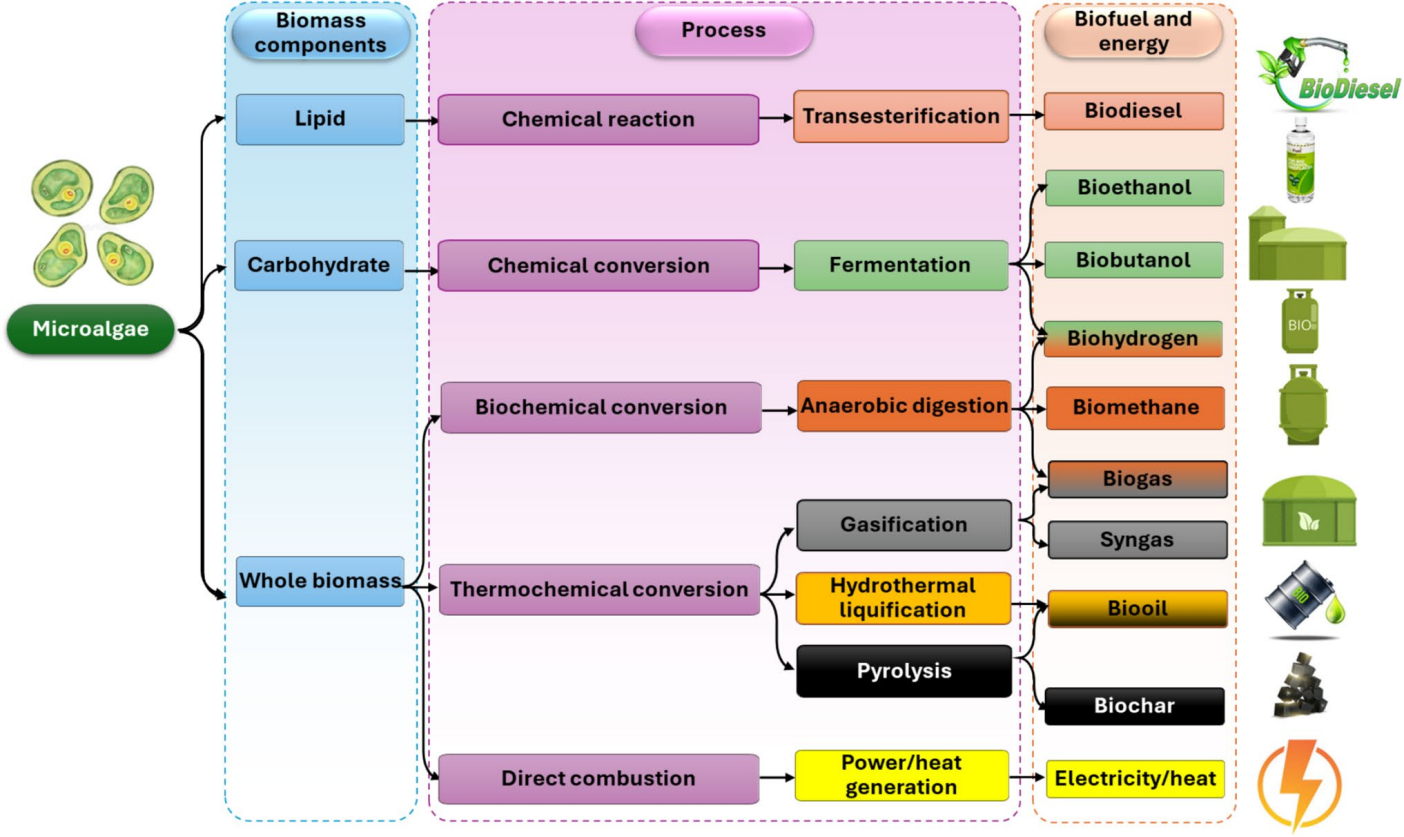
The conversion of microalgae into different biofuels

According to the World Oil Outlook Reports of Petroleum Exporting Countries (OPEC 2020), oil is expected to continue to be the most often utilized fuel in coming years despite the rapidly depleting oil reserves of OPEC members (Luna Loya 2021). However, due to the increasing oil prices, the dwindling natural oil resources, and the environmental impacts of fossil fuel use, using green algae as a feedstock for biofuel production has gained significant interest (EL-Seesy et al. 2022). Microalgae, as a third generation of biofuels, are recognized as the most promising feedstock for renewable biofuels due to their short generation time, rapid growth rate, high photosynthetic capacity, high lipid content, and low carbon footprint (Kumar et al. 2020a; EL-Seesy et al. 2022; Sallam et al. 2022). Additionally, biofuel generation from algae produces large quantities of oxygen and very low levels of sulfur, compared to fossil fuels, which release 29 million tons per year of CO_2_ and contribute 35.3 billion tons of CO_2_ emissions (Pandey 2017), contributing significantly to SDG 13 (climate action) and SDG 7 (affordable and clean energy). Microalgae may be utilized as a feedstock for various sustainable biofuels, including ethanol, biodiesel, biooil, and biohydrogen (Abomohra and Elshobary 2019; Kumar et al. 2020a), further supporting SDG 7 and SDG 12 (responsible consumption and production) as well as highlighting the potential for SDG 8 (decent work and economic growth) through the development of a new, sustainable industry. This versatility also indirectly contributes to SDG 9 (industry, innovation, and infrastructure) by fostering new technologies and industrial processes. The different processes for obtaining biofuels from microalgae are shown in Fig. 4.

#### Liquid biofuels

##### Biodiesel

Biodiesel from algae is one of the most promising types of algal biofuels. Microalgae can accumulate high levels of lipids, ranging from 20 to 50% of their dry weight, which can be used for biodiesel production (El-Sheekh 2021). Different algal species have been explored for their potential in biodiesel production, including *Chlorella, Dunaliella, Nannochloropsis*, and* Scenedesmus* (Abomohra and Elshobary 2019). The potential of *Skeletonema* in biodiesel production was also investigated (Gao et al. 2019; Wu et al. 2022). Life cycle analysis studies have shown that algal biodiesel can potentially have lower greenhouse gas emissions and a better energy balance compared to conventional diesel fuel (Collet et al. 2011).

Recent studies have highlighted the potential of algae as a source for biodiesel production, which emphasizes the eco-friendly nature of biodiesel and the need for alternative fuel sources (El-Sheekh et al. 2022b; Prajapati and Baraiya 2023; Osman et al. 2023).

##### Bioethanol

Bioethanol production from algae is an alternative approach to generating liquid biofuels from these versatile photosynthetic organisms. While algal biodiesel has received more attention, the potential to produce bioethanol from algae has also been explored. The process involves utilizing the carbohydrate content, primarily in the form of starch or cellulose, present in algal biomass (Ismail et al. 2020).

The production of bioethanol from algae typically involves several key steps. First, the algal biomass is pretreated to break down the cell walls and make the carbohydrates more accessible. This is followed by enzymatic hydrolysis, where enzymes such as amylases and cellulases convert the starch and cellulosic materials into fermentable sugars. These sugars are fermented using microorganisms, such as yeasts or bacteria, to produce bioethanol (El-Sheekh et al. 2022a).

One advantage of algal bioethanol is that it can be produced from the non-lipid fraction of the biomass, allowing for the co-production of valuable co-products like algal oils or other high-value compounds (Ashour et al. 2023). Additionally, certain algal species, such as *Chlorella* and *Chlamydomonas*, have been found to accumulate significant amounts of carbohydrates exceeding 50%, making them suitable feedstocks for bioethanol production.

Recent research has focused on the potential of algae as a source of bioethanol, a renewable and sustainable alternative to fossil fuels. (Anto et al. 2020) and (Ramachandra and Hebbale 2020) highlight the challenges and opportunities in this area, with Anto discussing the technological aspects of biofuel production from algae and Ramachandra specifically focusing on the potential of marine macroalgae. (Yew et al. 2019) provides a comprehensive overview of the entire process, from upstream cultivation to downstream processing, while (Khanna et al. 2019) explore the use of algae in the synthesis of metallic nanoparticles, which could have potential applications in bioethanol production. These studies collectively underscore the growing interest in and potential of algae-based bioethanol.

##### Biobutanol

Biobutanol production from algae is an emerging approach in the field of algal liquid biofuels. Biobutanol is an attractive alternative to bioethanol due to its higher energy density, lower hygroscopicity, and compatibility with existing gasoline infrastructure (Abomohra and Elshobary 2019). While research on algal biobutanol is still in its early stages, it presents promising opportunities for diversifying the range of biofuels derived from these versatile photosynthetic organisms (Pugazhendhi et al. 2019).

Producing biobutanol from algae involves utilizing the carbohydrate content, primarily in the form of starch or cellulose, present in the algal biomass. Like bioethanol production, the algal biomass is pretreated to break down the cell walls and make the carbohydrates more accessible. Enzymatic hydrolysis follows this, in which enzymes turn the starch and cellulosic materials into fermentable sugars.

However, the fermentation step for biobutanol production requires specialized microorganisms capable of producing butanol from fermentable sugars. Several bacteria, such as *Clostridium* species, have been explored for their potential in biobutanol fermentation from algal sugars (Pugazhendhi et al. 2019). Researchers have also looked into genetic engineering methods that could improve these microorganisms’ ability to make butanol when they use algal feedstocks (Abomohra and Elshobary 2019).

One benefit of algal biobutanol is that it can be used to make other valuable products, like algal oils or other high-value compounds, from the parts of the biomass that are not carbohydrates. Additionally, certain algal species with high carbohydrate accumulation could serve as promising feedstocks for biobutanol production (Shanmugam et al. 2021).

##### Biooil

Biooil or crude oil production from algae is a promising alternative to generating a liquid biofuel that can be refined into gasoline, diesel, and other transportation fuels. Hydrothermal liquefaction (HTL) (Guo et al. 2015) is the process by which the lipids, carbohydrates, and proteins (Guo et al. 2015) in the algae themselves are turned into crude oil through heat and pressure. The HTL process subjects the algal biomass to high temperatures and pressures in the presence of water, mimicking the natural geological processes involved in the formation of fossil fuels. In these conditions, the algal biomass goes through several complicated chemical reactions, such as depolymerization, decarboxylation, and deoxygenation, which creates crude oil (Guo et al. 2015).

One of the advantages of algal biooil production is its ability to utilize the whole algal biomass rather than just a specific fraction like lipids or carbohydrates. This maximizes the potential energy yield from the feedstock and reduces the need for extensive pretreatment or fractionation processes. The HTL process can also be applied to a wide range of algal species, including those with lower lipid content, expanding the potential feedstock options for biooil production (Sekar et al. 2021).

However, challenges remain in scaling up the HTL process for commercial biooil production from algae. These include developing efficient and cost-effective algal cultivation systems, optimizing the HTL process parameters for specific algal feedstocks, and upgrading and refining crude oil to meet transportation fuel standards. Supercritical CO_2_ and co-solvents have been investigated for producing biooil or crude oil from algal biomass as green, nonflammable, cheap, and eco-friendly, showing significant results compared to HTL (Patil et al. 2018).

Ongoing research efforts are focused on improving the overall efficiency and economics of algal biooil production and exploring the potential for co-product valorization from the byproducts generated during the process. With continued advancements, biooil from algae could contribute to a more sustainable and renewable energy future (Sekar et al. 2021).

#### Gaseous biofuels

While the focus on algal biofuels has primarily been on liquid fuels like biodiesel, bioethanol, and biooil, algae also hold the potential for producing gaseous biofuels, particularly biomethane and biohydrogen.

##### Biomethane

Biomethane, or renewable natural gas, can be produced from algal biomass through anaerobic digestion. In this process, algal biomass is broken down by specialized microorganisms in the absence of oxygen, resulting in the production of methane (CH_4_) and carbon dioxide (CO_2_) (Bose et al. 2020). The advantage of producing biomethane from algae lies in utilizing the entire algal biomass, including lipids, carbohydrates, and proteins, as feedstock for anaerobic digestion. This can maximize the energy yield from the algal feedstock compared to processes that only utilize specific fractions (Ma et al. 2021).

Algal species with high carbohydrate and protein content, such as *Chlorella* and *Spirulina*, are often considered promising feedstocks for biomethane production (Bose et al. 2020; Wu et al. 2020). Additionally, the residual biomass after lipid extraction for biodiesel production can be utilized for biomethane generation, enabling a biorefinery approach.

However, there are some problems with producing biomethane from algae. These include the need for effective pretreatment methods to make the algae biomass easier to digest, the need to find the best anaerobic digestion conditions for different types of algae feedstock, and the possibility of inhibitory compounds that can stop the digestion process (Ma et al. 2021).

##### Biohydrogen

Biohydrogen is created biologically using a range of microorganisms, including photosynthetic bacteria, dark fermentative bacteria, blue-green, and chlorophytes, in various metabolic routes (Bhatia et al. 2021). Recently, the importance of algae in biohydrogen generation has been extensively studied. Due to their excellent biomass production and photosynthetic activities, algae consume cheap inorganic compounds, adapt to varied water sources, and lack competition with arable land and high flue gas (CO_2_ and NO_x_) bioremediation, (Ubando et al. 2021). Several microorganisms can produce hydrogen, but cyanobacteria and green algae are the most well-known. These microorganisms are classified as third-generation feedstocks since they are more effective at transforming light energy into chemical energy and need less water and space for cultivation compared to other feedstocks (Sallam et al. 2022).

**Fig. 5:**
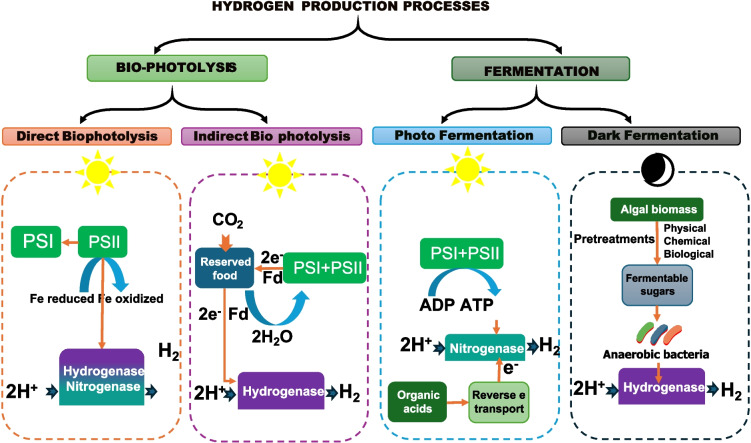
Hydrogen production from microalgae (A), direct biophotolysis (B), indirect biophotolysis (C), photo-fermentation processes (D), and dark fermentation processes (E). Modified with permission

There are two main ways to produce biohydrogen: light-dependent (direct or indirect biophotolysis) and light-independent (dark fermentation), illustrated in Fig. 5. Light-dependent biohydrogen production can be achieved through direct or indirect biophotolysis. Direct biophotolysis involves using photosynthetic microorganisms, such as green algae and cyanobacteria, to convert sunlight and water into hydrogen and oxygen. Indirect biophotolysis involves using photosynthetic microorganisms to produce an intermediate product, such as starch or lipids, which is then converted into hydrogen through fermentation. Light-independent biohydrogen production, also known as dark fermentation, involves the use of fermentative bacteria to convert organic matter, such as algal biomass, into hydrogen and carbon dioxide (Sallam et al. 2022; Rady et al. 2024).

To make green microalgae produce hydrogen, it is easiest to make the culture anaerobic by chemically or physically removing oxygen. This is because hydrogenase enzymes are sensitive to oxygen (Paramesh and Chandrasekhar 2020). This can be done by adding sodium dithionite solution or aerating with an inert gas like nitrogen or argon. Microalgae contain three forms of hydrogenases: nitrogenase, [FeFe]-hydrogenase, and [NiFe]-hydrogenase, with the latter two being more efficient for biohydrogen production. [FeFe]-hydrogenase is found in Chlorophyta like *Scenedesmus obliquus*, *Chlamydomonas,* and *Chlorella* (Wang et al. 2020). *Chlamydomonas reinhardtii, Chlamydomonas noctigama*, and *Chlamydomonas euryale* confirmed their ability to generate substantial hydrogen via biophotolysis under sulfur deprivation. *Scenedesmus obliquus* is another example that produces high hydrogen yields under anaerobic conditions with a continuous electron supply (Papazi et al. 2014).

Bacterial dark fermentation or oxygen-evolving photosynthesis are the most advanced biological ways to make H_2_ (Sallam et al. 2022; El-Sheekh et al. 2023a). Pre-treatment to break down microalgal cell walls and release fermentable sugars is crucial, involving methods like heat, acid/alkali, biological, grinding, sonication, pyrolysis, etc. (Sallam et al. 2022). Another approach is enzymes like nitrogenase or hydrogenase converting protons and electrons from water-splitting into H_2_ (Sharma and Arya 2017). The carbon/nitrogen (C/N) ratio is vital, where combining macroalgae like *Laminaria digitate* with microalgae like *Chlorella pyrenoidosa* and *Nannochloropsis oceanica* can optimize the C/N ratio for dark fermentation feedstock (Ding et al. 2016). Arthrospira platensis, with a low C/N ratio, can produce H_2_ via acid-treated biomass fermentation by microorganisms (Ding et al. 2016).

#### Bioelectricity

##### Dye-sensitized solar cells

**Fig. 6:**
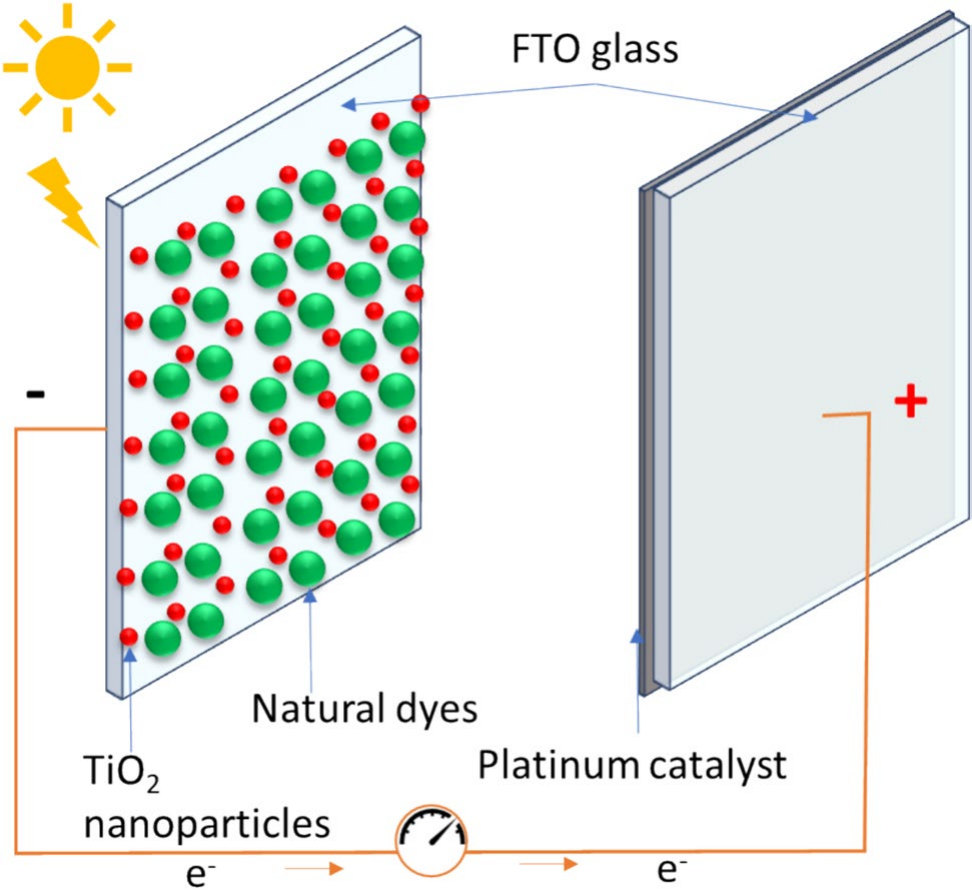
Diagram of a dye-sensitized solar cells reaction

Solar energy is considered clean and free, making it suitable for achieving sustainable development. Photovoltaic technology allows the conversion of sunlight into electricity. Photons in photovoltaic systems excite the electrons in the band known as valence to the higher energy band of conduction, where they are subsequently gathered and sent to an outer circuit (Al-Alwani et al. 2016). The first-generation solar cells are based on this technology and are made of monocrystalline silicon. Producing many harmful byproducts requires a challenging fabrication method and must be more eco-friendly (Belfar and Mostefaoui 2011). However, with conversion rates up to 24%, these cells offer the best efficiency (Calogero et al. 2015). The second generation of photovoltaic cells is based on thin films of amorphous semiconductors such as cadmium telluride (CdTe), amorphous silicon (a-Si), copper indium gallium selenite (CIGS), and recently, crystalline silicon (Ludin et al. 2014). The efficiency in these cells is increased due to the thin layers of deposited semiconductors, but the methods and processes involved in the rise in manufacturing cost. Despite this, the efficiency of silicon-based solar cells is greater (Al-Alwani et al. 2016). Third-generation solar cells are concentrated on maximizing efficiency and lowering production costs. Dye-sensitized solar cells (DSSC) comprise thin-film solar cells that utilize a dye to absorb sunlight and transform it into electrical power. In DSSCs, the photoelectrochemical cycle starts when a dye molecule that has been adsorbed on the surface absorbs a visible photon, this causes the dye to inject an electron into the TiO_2_’s conduction band, causing charge separation and the transportation of holes via the electrolyte (Fig. 6) (Tétreault and Grätzel 2012). In DSSCs, dyes play a significant role, and the solar cell’s effectiveness is greatly influenced by the dyes’ capacity to sensitize the solar spectrum and their efficacy at absorbing light. Nearly all synthetic dyes are harmful and expensive (Yu et al. 2014).

Recently, cheap, harmless natural dyes have been utilized as stimulants in dye-sensitive solar cells (Ludin et al. 2014). Chlorophylls, carotenoids, betalains, and anthocyanins are the main natural pigments used in solar cells (Narayan 2011; Narayan and Agrawal Pushpa 2012). These can be found in flowers, leaves, roots, and more recently, different bacteria and algae, such as micro- and macroalgae (Orona-Navar et al. 2020). The extraction process is relatively simple and less expensive compared to synthetic dyes. Natural dyes as photosensitizers in DSSC also present strong advantages like relative abundance, complete biodegradation, and environmental friendliness. Microalgae have received great attention because of their capability to produce photosynthetic pigments like chlorophylls and carotenoids with higher efficiency than terrestrial plants (Mohammadpour et al. 2014) (Fig. 6). Also, they can be produced at large scale.

In response to the brightness in the aquatic ecosystem, pigments from aquatic algae can display a variety of absorption ranges. In search of sensitizers with wide absorbance, the latter can be useful. Thus, compared to microalgal pigments, marine macroalgal pigments have undergone more extensive research as sensitizers.

##### Microbial electrochemical systems (MES)

Microbial electrochemical systems are novel systems employing microbial electrochemical phenomena to produce bioenergy or value-added chemicals. MES can be used for gathering rare earth elements from waste streams. MES includes MFC (microbial fuel cell), SMFC (sediment microbial fuel cell), MEC (microbial electrolysis cell), MDC (microbial desalination cell), MRC (microbial reverse-electrodialysis cell), MESC (microbial electrosynthesis cell), etc. (Wang and Ren 2013). Microalgae coupled MES assist bioelectricity and industrial supplies production along with the elimination of pollutants from wastewater. MFCs consist of an anode and cathode chamber where the microbial community facilitates electron flow, converting chemical energy into electricity. This technology, which has evolved over the last few decades, can generate high power densities when using substrates like carbohydrates and organic waste (Elshobary et al. 2021).

MFC configurations vary, typically being dual-chambered or single-chambered, which affects their efficiency. Single-chamber reactors, often used with species like *Chlorella vulgaris*, rely on photosynthesis for power generation. Dual-chamber designs use algae at both the cathode and anode for optimal energy production. Some configurations integrate sediment layers, enhancing both electricity and oxygen generation as well as desalination seawater (Sharma et al. 2019).

Although promising, scaling BES technology faces challenges, such as optimizing algae strains for bioenergy output and integrating BES into wastewater treatment at larger scales. Continuous improvements in system configuration, microbial selection, and operational modes are needed to increase efficiency and feasibility.

### Bioplastics

Plastics are categorized by origin (fossil or biobased) and biodegradability (non-biodegradable or biodegradable) (Devadas et al. 2021; Zanchetta et al. 2021). Fossil-based plastics pollute the environment, taking centuries to degrade (Senousy et al. 2023). Since the 2000s, a growing focus has been on developing bioplastics from renewable plant materials as eco-friendly alternatives (Singh et al. 2022). Bioplastics are made fully/partly from biomass, like crops, and function like petroleum-based plastics (Elkaliny et al. 2024). Global bioplastics production was 2.15 million tons, projected to rise to 2.87 million tons by 2025 (European Bioplastics 2021).

Demand for advanced biopolymers like polyhydroxyalkanoates (PHAs) is increasing. The PHA market was valued at over $85 million in 2021 and is expected to grow at over 10.5% CAGR between 2022 and 2030, driven by its use in food packaging. Biobased polypropylene, introduced commercially in 2019, has significant growth potential through 2024 due to wide industry applications (Siracusa and Blanco 2020).

#### Classification of synthetic polymers and bioplastics

**Fig. 7:**
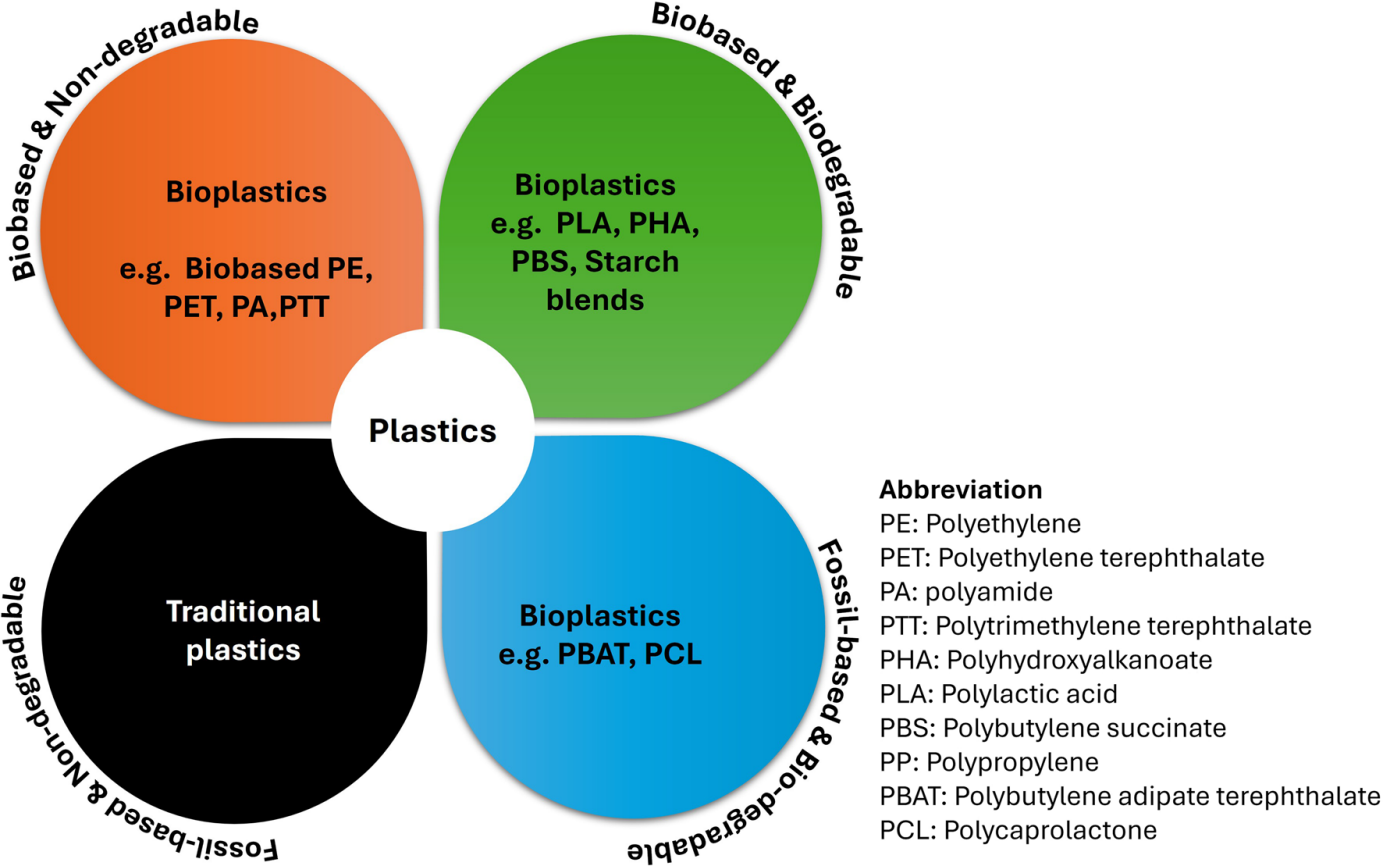
Plastics classification corresponding to biodegradability and biobased content

Bioplastics can be categorized into four quadrants based on their origin (bio or fossil-based) and biodegradability (European Bioplastics 2021), as shown in Fig. 7. The second quadrant includes biobased and biodegradable polymers like PBS and PHA, PLA, and starch blends. Plants and microalgae are promising sources for bioplastics, containing starch and cellulose used as raw materials (Zanchetta et al. 2021). Microalgae are rich in bioactive compounds like lipids, fatty acids, PHA, carbohydrates, proteins, and cellulose, making them suitable for bioplastics production (Avargani et al. 2022).

Current methods for producing microalgae-based bioplastics include (i) obtaining cellulose/starch directly from biomass, (ii) using microalgae for PHAs/PHB production via natural or genetically modified organisms, (iii) blending with synthetic polymers to enhance properties, (iv) combining microalgae biomass with bioplastics like PLA, and (v) producing PLA from microalgae (Porse and Rudolph 2017; Lim et al. 2021). All of these contribute to SDG 12 (responsible consumption and production) and SDG 9 (industry, innovation and infrastructure). PHAs are promising bioplastic alternatives to petroleum plastics, being biocompatible, and biodegradable with unique properties like UV resistance, supporting SDG 14 (life below water) and SDG 15 (life on land) by reducing plastic pollution. Microalgae naturally produce PHAs under nutrient limitation (Devadas et al. 2021). Bioplastics undergo microbial, aerobic, or anaerobic biodegradation, e.g., PHA degrades in 5–6 weeks aerobically, 3 weeks anaerobically, and 18 days via microbial action (Emadian et al. 2017; Abraham et al. 2021), further contributing to SDG 11 (sustainable cities and communities) by addressing waste management challenges and SDG 3 (good health and well-being) by reducing environmental pollution from persistent plastics.

#### Bioplastics applications

Applications for bioplastics include electronics, packaging, construction, textiles, shopper goods, and medical tools and pads. European Bioplastics states that bioplastics are extensively employed in the packaging industry, particularly rigid and flexible packaging, textiles, and other products (European Bioplastics 2021). Packaging dominates the other market sector, with 53% (1.14 million tons) of the entire bioplastic market in 2019 (Siracusa and Blanco 2020). The use of bioplastics is rising across every sector of the economy due to customers’ increasing demand for sustainable products. Then, 55.5% of the 2.11 million tons of bioplastics produced can be categorized as sustainable or biodegradable (such as 13.9% polylactic acid, 4.3% poly-butylene succinate, 1.2% PHA, and 1.4% other substances, as well as 21.3 starch blends) and biobased/non-biodegradable is the classification for the remaining 44.5% of bioplastics (such as 11.8% polyethylene, 11.6% polyamides, 9.8% polyethylene terephthalate, 9.2% polypropylene terephthalate, 0.1% polyethylene furanoate, 0.9% polypropylene, and 1.1% others) (European Bioplastics 2021). On the other hand, PHAs have a wide range of uses, including packaging films, containers, supermarket bags, fiber and foam substances, cutlery, cups, drug delivery, medical devices, carriers, etc.

#### Favorable microalgae strain for bioplastic production

It was reported that a variety of microalgae species can sequester and retain PHA within their cells, such as* Arthrospira platensis*, *Dunaliella tertiolecta*, *Chlorella vulgaris, Scenedesmus* sp., and *Synechococcus* sp. (de Farias Silva and Sforza 2016). These species are typically cultured under nitrogen and phosphorus deficiency. A vital nutrient for microalgae growth is nitrogen, which is responsible for 10% of the biomass of these algae when combined with ammonium and nitrate (Razzak et al. 2017). When microalgae do not have enough nitrogen, they focus on taking in nitrogen molecules so they can store neutral lipids, especially triacylglycerides (TAGs) and PHAs, which help them stay alive (Chu et al. 2013). Limiting nitrogen can increase lipid concentrations by up to 85% and reduce biomass production.

Tiny cells and high *Chlorella* and* Arthrospira* protein levels make them appropriate for bioplastic conversion without pre-treatment processes, allowing for cost-effective, large-scale manufacturing with reduced waste output. This makes them favorable for the production of sheets and fabrics*.* (Zeller et al. 2013). It was found that *Botryococcus* can contain 60% proteins, 30% carbohydrates, and 47% lipids, similar to crude oil (Cabanelas et al. 2015), while *Chlamydomonas* can produce up to 21%, 48%, and 17% of its dry weight in lipids, proteins, and carbohydrates, respectively (Shuba and Kifle 2018). These species are potential candidates for bioplastic production in the future (Table 2).

**Table 2:** Microalgae species contribution to bioplastic

Microalgae	Biopolymer	Characteristics	Reference
*Arthrospira*	Bioplastic film & PHA	46–63 (wt%) protein	(Dianursanti et al. 2019; Afreen et al. 2021)
*Nostoc muscorum*	PHAs	High protein content, cellulose & starch	(Afreen et al. 2021)
*Chlamydomonas*	Starch-based bioplastics	49% (w/w) starch	(Mathiot et al. 2019)
*Chlorella vulgaris*	PVA & PHB	51–58 (dw%) protein	(Selvaraj et al. 2021)
*Botryococcus braunii*	Ultrafine fibers by electrospinning	High protein content	(Verdugo et al. 2014)
*Synechocystis PCC6803* *Synechococcus MA19*	PHAs	High protein content, cellulose & starch	(Afreen et al. 2021)
*Dunaliella tertiolecta*	PHAs	High lipid content	(Costa et al. 2019)
*Phaeodactylum tricornutum*	PHB	PHB levels of up to 10.6% of the dry algal weight	(Hempel et al. 2011)
*Hydrodictyon reticulum*	Polylactic acid (PLA)	47.5% reducing sugars	(Nguyen et al. 2012)

### Therapeutic potentials

Microalgae are increasingly recognized as valuable sources of bioactive compounds like proteins, polysaccharides, fatty acids, vitamins, sterols, phenolics, phycobilins, carotenoids, and enzymes, offering opportunities for pharmaceuticals and cosmetics (Chu and Phang 2019). This diversity of compounds contributes to SDG 3 (good health and well-being) by providing potential new sources for medicine and health products. The development of these resources aligns with SDG 9 (industry, innovation, and infrastructure) by fostering innovation in biotechnology. Additionally, the sustainable production of these compounds supports SDG 12 (responsible consumption and production) by offering alternatives to synthetic or environmentally harmful ingredients. The potential for new industries based on these bioactive compounds also contributes to SDG 8 (decent work and economic growth) by creating job opportunities in research, production, and related sectors. Furthermore, as these compounds can be derived from sustainable sources, they indirectly support SDG 14 (life below water) and SDG 15 (life on land) by reducing reliance on terrestrial or marine resources that may be under pressure from overexploitation.

**Fig. 8:**
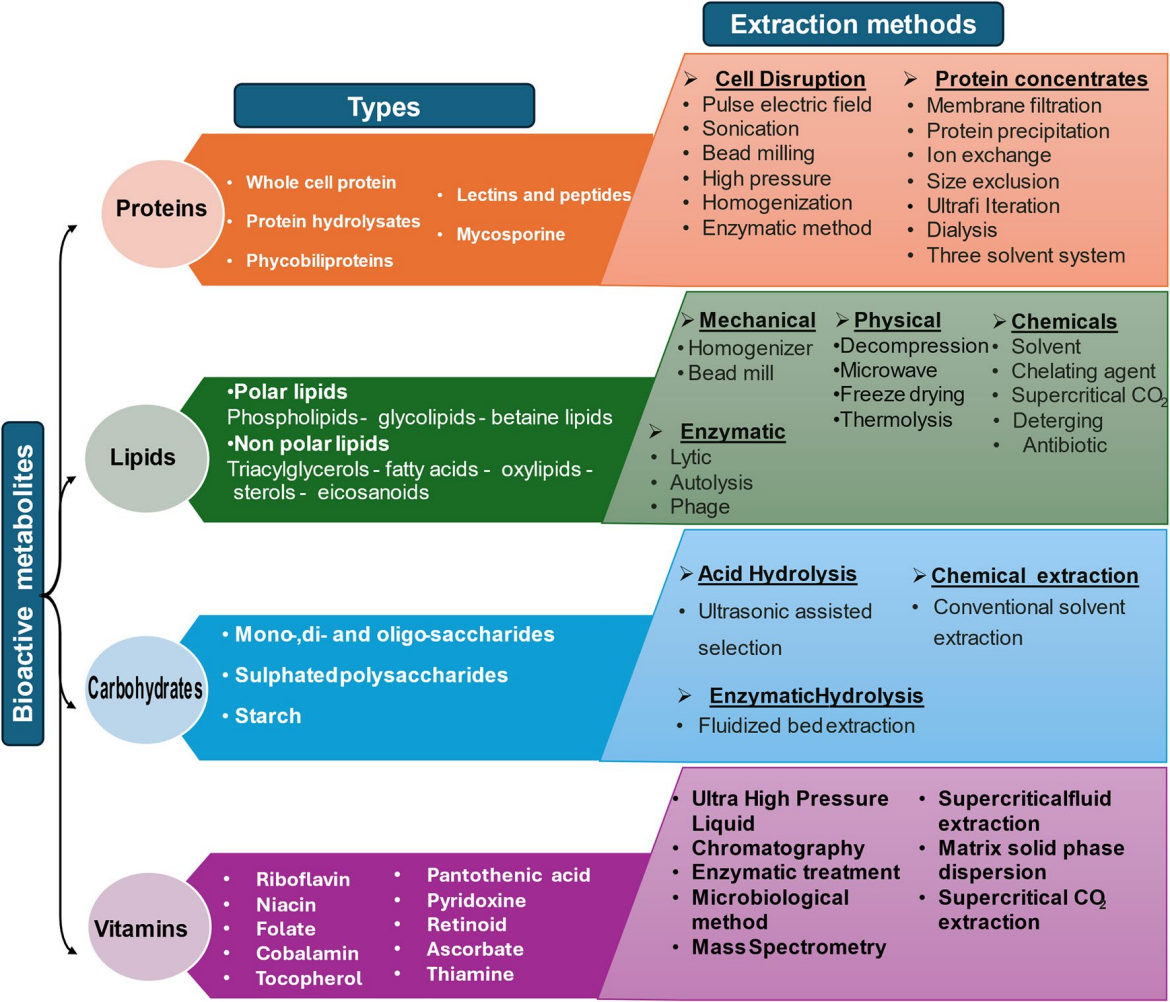
Microalgae’s biomass bioactive compounds and their extraction methods

Each of these bioactive metabolites has its extraction methods. Proteins are extracted through cell disruption techniques like sonication and high-pressure homogenization, or concentration methods such as membrane filtration and chromatography. Lipids are extracted via mechanical methods like homogenization, enzymatic processes, or chemical solvents including supercritical CO_2_. Carbohydrates are obtained through acid hydrolysis, chemical extraction, enzymatic hydrolysis, or fluidized bed extraction. Vitamins are extracted using advanced techniques such as ultra-high-pressure liquid chromatography, enzymatic treatment, and mass spectrometry-based methods (Fig. 8). These bioactive components can be extracted separately or in the sequencing process to maximize the benefits and reduce production costs. This diverse array of compounds and extraction techniques underscores the versatility and potential of microalgae in various applications, from nutraceuticals to pharmaceuticals.

In the 1970s, *Chlorella* preparations were used to treat cervical and vaginal irritation, leading to algae-based drugs in Eastern Europe (Honĕk et al. 1978). Recent interest focuses on algae’s technological development and use in treating diseases as an eco-friendly, low-cost, and abundant material (Katz and Baltz 2016).

*Chlorella* supplements have proven beneficial for cardiovascular conditions, improving fat and glucose metabolism and altering gene expression for reduced blood sugar levels (Mizoguchi et al. 2008). *Chlorella vulgaris* tablets improved blood sugar control, reduced inflammation, and enhanced liver function in non-alcoholic fatty liver disease (Ebrahimi-Mameghani et al. 2017). *C. vulgaris* and *Chlamydomonas pyrenoidosa* exhibit antimicrobial activities (Jayshree et al. 2016). *Dunaliella salina* produces beta-carotene with anti-inflammatory and immunomodulatory properties, potentially useful against prostate and colon cancer (Kumar et al. 2020c). *Arthrospira platensis* impacts hemopoiesis, antibody, and cytokine production and exhibits antiviral, antifungal, antibacterial, anticancer, and anti-inflammatory properties (Balasubramaniam et al. 2022). Microalgae show therapeutic benefits against skin cancer and cardiovascular disease and the potential for antiaging skincare (Balasubramaniam et al. 2022).

Advances include diatom microalgae-based drug delivery systems loaded with anticancer complexes, improving colorectal cancer cell adherence and controlled drug release, and reducing dosage and side effects. Microalgae-derived drugs offer increased efficacy, reduced toxicity, and improved absorption, potentially improving patient quality of life (Delasoie et al. 2020).

#### Anticancer activity

Unhealthy lifestyle choices, such as poor diet, lack of physical activity, and exposure to harmful substances like tobacco and alcohol, can increase the risk of developing cancer in individuals of all ages, including children. While chemotherapy and radiotherapy are commonly used cancer treatments, they can have side effects, and the chances of recovery depend on various factors, including the type and stage of cancer, as well as the individual's overall health. Researchers are exploring the potential of bioactive compounds found in microalgae, such as polysaccharides, fucoxanthin, carotenoids, peptides, proteins, and lipids, for their potential chemopreventive and anticancer properties. However, more research is needed to fully understand their efficacy and safety (Talero et al. 2015). Recent research has examined the possible anticancer effects of algal meroterpenoids (AMTs, 1–8) isolated from Phaeophyceae *Cystoseira usneoides*. By reducing protein kinase B (AKT) phosphorylation levels in colon cancer cells, it was found that these AMTs had low toxicity against normal colon cells and effectively inhibited the proliferation of HT-29 cancer cells (Zbakh et al. 2020). Other examples are shown in Table 3.

**Table 3:** The most important species that have role in medical applications

Applications	Properties	Bioactive compounds	Algae	References
Cosmetics	Antioxidant, moisturizing, antiaging, UV screening or antitanning	Crude extracts	*Chondrus crispus, Ascophylum nodosum, Alaria esculenta, Arthrospira platensis, Nannochloropsis oculata, Porphyra haitanensis, Scenedesmus quadricauda, Chlorella vulgaris*, & *Dunaliella salina*	(Joshi et al. 2018; Khan et al. 2020)
Pharmaceuticals	Antibacterial	Crude extracts	*Chlorella vulgaris, Chlamydomonas pyrenoidosa*	(Jayshree et al. 2016)
	Useful toxin	Toxins	*Ochromonas* sp*., Prymnesium parvum*	(Katircioglu et al. 2006)
	Anticancerous	Cryptophycin	*Nostoc* sp.	(Eggen and Georg 2002)
	Antiproliferative and anti-inflammatory	Scytonemin protein	*Stigonema* sp.	(Rastogi et al. 2015)
	Anticancerous	Crude extract	*Chaetomorpha *sp.	(Haq et al. 2019)
	COVID‑19 treatment	Astaxanthin	*Haematococcus pluvialis, Nostoc ellipsosporum*, and *Arthrospira*	(Rosales-Mendoza et al. 2020; Singh et al. 2020; Talukdar et al. 2020)
	Antihypertensive & antihyperlipidemic activity	Crude extract	*Nannochloropsis, Arthrospira, & Isochrysis*	(Chen et al. 2020)
Dentistry	Strengthens osteoblastic and reduces osteoclastic activity	Astaxanthin	*Microalgae*	(Balci Yuce et al. 2018)
	Reduced probing pocket depth and enhanced clinical attachment level in chronic periodontitis	*S. platensis* gel	*S. platensis*	(Elgendy et al. 2024)
	Reduced alveolar bone loss in periodontitis	Fucoxanthin	Diatoms	(Kose et al. 2016)

#### Anti-inflammatory effect

Microalgae extracts have been found to possess anti-inflammatory properties (Rocha et al. 2022) (Table 3). Extracts from *Chlorella* sp. WZ13 was shown to reduce nitrite production, inhibit the expression of inducible nitric oxide synthase (iNOS) protein, and decrease the production of inflammatory cytokines in lipopolysaccharide (LPS)-stimulated cells (Yang et al. 2022). Similarly, extracts from *Haematococcus pluvialis*, *Nannochloropsis oceanica*, *Tisochrysis lutea*, and *Porphyridium cruentum* had high antioxidant and anti-inflammatory capacities (Sharma et al. 2023). These microalgae extracts exhibited moderate and selective cholinesterase inhibitory potential and the ability to inhibit the release of proinflammatory cytokines (Agatonovic-Kustrin and Morton 2022). The anti-inflammatory effects of microalgae extracts make them promising candidates for developing therapeutic agents against inflammation-related disorders (Gallego et al. 2022).

#### Antiviral activity

It was reported that cyanobacteria, dinoflagellates, and rhodophytes have antiviral properties. Sulfated polysaccharides of microalgae have the strongest antiviral properties, where it has virus replication inhibitory activity such as flavivirus, arenavirus, herpesvirus, togavirus rhabdovirus, and orthopoxvirus groups, in sometimes, sulfate groups can combine with calcium ions to form molecules that have antiviral properties (Kaparapu et al. 2022). Microalgal polysaccharides have been shown to exhibit antiviral activities by inhibiting viral entry and adsorption into host cells. Specifically, microalgal extracts containing compounds such as fucoidan, laminarin, and alginate have demonstrated potential in combating viral infections (De Jesus Raposo et al. 2015). The microalgal species that have been particularly studied for their antiviral properties include *Arthrospira* and *Chlorella* (De Jesus Raposo et al. 2015), as well as other species mentioned in Table 3.

#### Antioxidant activity

As the human body cannot synthesize antioxidants, microalgae pigments benefit human health (De Jesus Raposo et al. 2013) (Table 3). Antioxidants play a crucial role in reducing the damage caused by free radicals in the human body, which can lead to oxidative stress and various chronic diseases (Gaete Olivares et al. 2016). Microalgae are rich in various antioxidant compounds, including carotenoids (such as astaxanthin, lutein, and zeaxanthin), phenolic compounds, vitamins (e.g., vitamin E), and enzymes (e.g., superoxide dismutase and catalase) (Sathasivam et al. 2019). These antioxidants can help lower the risk of developing chronic diseases involving cancer, cardiovascular diseases, neurodegenerative disorders, and age-related macular degeneration (De Jesus Raposo et al. 2013).

Carotenoids, in particular, are potent antioxidants that can neutralize free radicals and reactive oxygen species, thereby protecting cells from oxidative damage (Sathasivam et al. 2019). Astaxanthin, a carotenoid found in microalgae such as *Haematococcus pluvialis* and *Chlorella zofingiensis*, has been extensively studied for its strong antioxidant activity and potential health benefits, including anti-inflammatory, neuroprotective, and anticancer effects (Ambati et al. 2014; Shah et al. 2016).

The incorporation of microalgae or their extracts rich in antioxidants into functional foods, nutraceuticals, and dietary supplements holds promising potential for promoting human health and preventing chronic diseases associated with oxidative stress (De Jesus Raposo et al. 2013; Basheer et al. 2020).

#### Gut health

The prebiotic quality of microalgae and their higher fiber and carbohydrate contents make them beneficial for gut health. Prebiotics are non-digestible food ingredients that selectively stimulate the growth and activity of beneficial gut bacteria, thereby promoting a healthy gut microbiome (Gibson et al. 2017). Microalgae can improve gut health by providing sustainable protein sources and functional food ingredients. They offer several advantages over animal-derived proteins, including lower risk of chronic diseases such as heart disease, non-alcoholic fatty liver disease (NAFLD), and inflammatory bowel disease (IBD) (Eilam et al. 2023). Microalgae are rich sources of various prebiotic compounds, including polysaccharides, oligosaccharides, and dietary fibers (De Jesus Raposo et al. 2016; Gouda et al. 2022).

The human gut microbiome plays a crucial role in maintaining overall health and offers protection against various diseases (Lin et al. 2014). A diverse and balanced gut microbiota is associated with improved digestion, nutrient absorption, immune function, and reduced risk of chronic diseases such as inflammatory bowel diseases, obesity, and metabolic disorders (Nagpal et al. 2016).

Microalgae-derived prebiotics can selectively promote the growth of beneficial gut bacteria, such as Bifidobacterium and Lactobacillus species, which are known to produce short-chain fatty acids (SCFAs) and other metabolites that contribute to maintaining gut homeostasis and overall health (Tiwari and Troy 2015; De Jesus Raposo et al. 2016). These prebiotic compounds can also modulate the immune system, reduce inflammation, and enhance the bioavailability and absorption of essential nutrients.

Additionally, microalgae contain many bioactive compounds, such as polyphenols, carotenoids, and polyunsaturated fatty acids (PUFAs). These can work together to improve gut health by acting as antioxidants, reducing inflammation, and changing the immune system (Costa et al. 2020; Geada et al. 2021).

The prebiotic potential and functional properties of microalgae make them promising candidates for the development of functional foods, nutraceuticals, and dietary supplements aimed at promoting gut health and preventing gut-related disorders (Tiwari and Troy 2015; De Jesus Raposo et al. 2016).

#### Skin treatment

Microalgal bioactive compounds have promising applications in treating various gynecological and skin diseases, including dermatitis and lupus (Khavari et al. 2021). In a study on the therapy of skin cancer, astaxanthin (a microalgae-derived carotenoid) loaded onto cellulose nanocrystals/nanofibrils demonstrated the potential to modulate intracellular signaling pathways in cancer cells and promote apoptosis (programmed cell death) of skin cancer cells (Shanmugapriya et al. 2020).

Carotenoids, potent natural antioxidants in microalgae, have gained attention in the cosmetics industry for their antiaging properties in skin care products. They have also been shown to reduce dandruff, improve skin hydration, restore flexibility, and treat dry or damaged skin (Aslam et al. 2021; Zhuang et al. 2022).

Table 3 summarizes the potential applications of various microalgal carotenoids, such as astaxanthin, lutein, and fucoxanthin, in skincare and cosmetic products. These carotenoids exhibit antioxidant, anti-inflammatory, and photoprotective properties, making them suitable for formulations targeting skin aging, hyperpigmentation, and sun damage.

It is important to note that while these studies demonstrate the potential of microalgal bioactive compounds in treating skin and gynecological conditions, further research is necessary to fully understand their mechanisms of action, optimal dosages, and safety profiles for human use. Additionally, more clinical trials may be required to validate their efficacy before they can be widely incorporated into therapeutic or cosmetic products.

#### Dentistry

Microalgae use in dentistry is in its early stages. Evidence has shown that microalgae exhibit potent oral antiviral, anti-inflammatory, antibacterial, antifungal, and anticancer activity in the oral cavity (Balasubramaniam et al. 2022). Toothpaste can demonstrate antimicrobial activity by incorporating 1% of iota carrageenan, blue-green algae (*Aphanizomenon flos-aquae*), and *Laminaria japonica*. Algal extracts with antimicrobial properties are suitable for developing oral hygiene products such as toothpaste, mouthwash, and chewing gum. Additionally, polysaccharides derived from algae can create gels with shape memory capabilities, making them ideal for making dental impression materials. The study on obtaining new pharmaceutical formulations based on mixtures of collagen gels and extracts from marine algae discussed the incorporation of hydroalcoholic extracts from marine algae into collagen matrixes for regenerative therapy (Kiziltan et al. 2015). The study also mentioned the potential nanomaterial properties of these formulations, acting at trans-dermal and transmucosal levels. Microalgae-derived components can serve as carriers for bisphosphonate drugs, which are commonly used to treat bone-related disorders such as osteoporosis, bone metastasis, and myeloma. While oral consumption of silica materials containing bisphosphonate drugs has been associated with certain adverse effects, a novel strategy has emerged that addresses this issue. Researchers have successfully incorporated bisphosphonate-sodium alendronate into the biosilica shells of the diatom *Thalassiosira weissflogii* (Cicco et al. 2019) (Table 3).

#### COVID‑19 treatment

Since coronavirus disease (COVID-19) is currently the most significant health concern in the world, finding effective treatments for this condition is urgently needed to prevent thousands of deaths (Talukdar et al. 2020). One of the main causes of death for COVID-19 patients is severe respiratory pain syndrome (ARDS), a condition linked to cytokine storm syndrome. According to Yang et al. (2020), proinflammatory cytokines (IL-1, IL-6, and TNF-α) and chemokines (CCL2, CCL3, CXCL9, and CXCL10) are produced in more significant amounts during a cytokine storm, leading to immune system hyperactivity and severe lung injury (ALI). Carotenoid has several medicinal uses, such as astaxanthin, which also possesses immunomodulatory, anti-inflammatory, and potent antioxidant properties. A microalga known as *Haematococcus pluvialis* is particularly rich in astaxanthin. Recent studies have indicated that supplementing with astaxanthin in individuals diagnosed with COVID-19 may help mitigate the severity of cytokine storms, a dysregulated immune response that can potentially lead to acute respiratory distress syndrome (ARDS) and acute lung injury. The anti-inflammatory and immunomodulatory effects of astaxanthin could play a crucial role in regulating the excessive inflammatory response associated with severe COVID-19 cases, thereby reducing the risk of developing ARDS and other respiratory complications (Talukdar et al. 2020) (Table 3). Specified mono- and oligosaccharides can be bound by proteins called lectins. Lectin, known as cyanovirin-N, which has been shown to have antiviral properties against the influenza virus, was isolated from the cyanobacterium *Nostoc ellipsosporum*. Furthermore, Carrageenan, as sulfated polymer derived from microalgae, can prevent viruses from adhering to, reproducing, and transcribing in host cells (Rosales-Mendoza et al. 2020).

In a previous study, *Arthrospira* had potent antiviral activities. Polysaccharides obtained from *Arthrospira* inhibit the replication of numerous viruses, such as influenza (Singh et al. 2020). Furthermore, *A. platensis* could improve the immune system’s defenses against viruses by inducing immune cell activation and the production of interferon-gamma, an essential cytokine possessing antiviral capabilities. A pigment derived from *Arthrospira*, phycocyanin also has anti-inflammatory properties and is a NADPH oxidase inhibitor. *Arthrospira* in particular seems to be a potential candidate for adjuvant treatment of COVID-19 patients (Singh et al. 2020).

#### Microalgal-based nanocarriers in drug delivery

Systems for delivering medications or genes to target cells, like cancer cells, are known as drug delivery systems. A damaged or absent genome is frequently present in patients with genetic diseases. Silica nanoparticles (NPs) are thought to be an efficient method for delivering genes in this situation (Dolatabadi and de la Guardia 2011). Modern medicine delivery systems can surpass traditional medications’ disadvantages (high toxicity and poor solubility/stability) by getting the medication into the appropriate body tissues. Micelles, silicon oxide nanoparticles (NPs), and liposomes are common drug transporters; each has advantages and disadvantages. As for cons of these NPs, their synthesis is expensive, time-consuming, needs a lot of energy, and uses harmful components (Aw et al. 2012; Maher et al. 2018). Alginate, carrageenan, laminarin, and fucoidan are examples of polysaccharides that microalgae can make. These materials can be converted into nanoparticles (NPs), which interact with biomolecules through hydrophilic structures on their surfaces (Shankar et al. 2016). Diatoms are inexpensive and easy to grow and have an amorphous silicate shell. Another source of porous silica (SiO_2_) NPs is diatomaceous earth (DE)/frustules. Conversely, diatom shells are used to create nanoparticles (NPs) that are utilized for carrying drugs and biomolecules because of their unique three-dimensional forms. Drug loading and release from DE NPs may be enhanced by changing the shape and the functionalization process (Sasirekha and Santhanam 2019). Natural silica nanoparticles from the diatom *Coscinodiscus concinnus* were utilized to deliver the hydrophilic antibiotic streptomycin (Gnanamoorthy et al. 2014). They found that the treated diatoms’ drug release efficiency time was longer than untreated diatoms. Furthermore, due to surface adsorption, streptomycin was taken up by the pores and the hollow diatom structure. In several studies, modified DE NPs have been studied for the targeted administration of medicines (paclitaxel and camptothecin) in the management of breast and colon tumors, with promising outcomes. Clinical studies and additional research are still needed in this area.

### Food security

Microalgae contribute significantly to SDG 1 (no poverty), SDG 2 (zero hunger), and SDG 3 (good health and well-being) by offering a sustainable and nutritious protein source. Microalgal protein exhibits great digestion in dietary supplements and is a dependable and sustainable supply. Enzymatic hydrolysates of microalgae can be used in nutritional health formulae in place of traditional protein sources (such as seafood protein, milk protein, and soybean) (Kose et al. 2017). This innovation aligns with SDG 9 (industry, innovation, and infrastructure) by fostering new food technologies and production methods.

*Chlorella vulgaris*, which is rich in selenium, can be used as a source of microalgae as a trace element supplement. It was found that Se-*Chlorella* has a higher bioavailability rate of 49% when compared with selenium yeast (21%), various types of supplements (32%), and foods containing selenium, according to their analysis of accessibility data (Vu DaiLong et al. 2019). This enhanced bioavailability contributes to SDG 3 by improving nutritional outcomes and potentially reducing micronutrient deficiencies.

The cultivation of microalgae for food and nutritional purposes also supports SDG 12 (responsible consumption and production) by offering a resource-efficient alternative to traditional agriculture. Moreover, it indirectly contributes to SDG 13 (climate action) and SDG 14 (life below water) by reducing pressure on marine ecosystems for protein sources and potentially lowering greenhouse gas emissions associated with conventional livestock farming.

Additionally, the development of microalgae-based food industries can promote SDG 8 (decent work and economic growth) by creating new job opportunities in the research, production, and distribution sectors. This emerging field also has the potential to address SDG 10 (reduced inequalities) by providing accessible, high-quality nutrition sources to diverse populations, including those in food-insecure regions.

#### Meat processing

Algae used to be applied as a food ingredient in meat-processing facilities to enhance shape and flavor (Matos et al. 2022). Algae with a high protein content have emerged as a novel source of superior protein with the advent of animal substitutes. Nutritionists nowadays agree that *Arthrospira* is a great source of natural protein food, with a protein concentration of 60–70% and a human absorption rate of up to 95% (AlFadhly et al. 2022). *Arthrospira* and *Chlorella* are widely utilized by researchers to create meat substitutes (Michel et al. 2021).

#### Drinks

Microalgae, such as *Spirulina, Chlorella*, and *Dunaliella salina*, are increasingly being used in drinks due to their nutritional value and potential health benefits. These tiny algae are packed with protein, vitamins, minerals, antioxidants, and omega-3 fatty acids. Adding microalgae to drinks can enhance hydration, provide sustained energy, protect against oxidative damage, and reduce inflammation (Kumar et al. 2023). A recent study found that consuming a spirulina-enriched beverage improved hydration status and reduced oxidative stress in healthy adults. Another study showed that a chlorella-containing drink enhanced energy levels and cognitive function in healthy volunteers (Sherafati et al. 2022).

Microalgae can be incorporated into a variety of drinks, including smoothies, juices, energy drinks, functional beverages, and water enhancers. They can add distinctive flavors, textures, and colors to drinks, making them both nutritious and visually appealing. When using microalgae in drinks, it is important to consider the taste, texture, and dosage. Some microalgae species may have a strong flavor that needs to be balanced with other ingredients. Additionally, certain species can create a gritty texture, which may require blending or straining. The recommended dosage of microalgae in drinks varies depending on the species and desired benefits. It is also important to source high-quality microalgae from reputable suppliers to ensure safety and nutritional value.

#### Systems for aquaponics using blue-green algae

Cyanobacteria have long been used as inoculants in rice fields to fix nitrogen and increase yields. With the projected 60–100% rise in food demand by 2050, aquaculture has become crucial for twenty-first century food security (Market Research Report 2020). Fish farming is rapidly growing, expected to increase by over 7.1% between 2020and 2027 (Market Research Report 2020). Over 68% of commercial fish and shrimp species rely on protein-enriched fish feed (Tacon 2020; Chen and Wang 2021), traditionally using fish meal. However, fish meal market fluctuations have impacted aquaculture profitability (Jannathulla et al. 2019), necessitating alternative feed ingredients that are economically viable, environmentally sustainable, nutrient-rich, easily digestible, palatable, and avoid heavy metals, fibers, non-soluble carbohydrates, and antinutritional components (Nagappan et al. 2021).

Microalgae have been proposed as an alternative fish feed, offering a sustainable and potentially economically viable source. Algae like *Pavlova, Tetraselmis, Nannochloropsis, Skeletonema, Phaeodactylum, Thalassiosira, Arthrospira*, and *Chlorella* are preferred for aquaculture feed, with high-protein algae like *Cryptonemia crenulata* and *Hypnea cervicornis* for shrimp (Kaur and Singh 2024). Fish hatcheries use *Dunaliella* sp., *Isochrysis* sp., *Arthrospira* sp., and *Pavlova* sp. as larvae feed feed (Hodar et al. 2020). *Nannochloropsis oculata* and *Schizochytrium* sp. improved health in Nile tilapia (Souza et al. 2020), while *Nannochloropsis* sp. and *Isochrysis* sp. enhanced nutrient digestibility in rainbow trout (Sarker et al. 2020).

Algae are rich in essential nutrients like polyunsaturated fatty acids (PUFAs), proteins, and pigments beneficial for aquaculture feed. Microalgae produce PUFAs like arachidonic acid, eicosapentaenoic acid, and docosahexaenoic acid, enhancing fish growth and immunity (Chen et al. 2019). Microalgae contain 40–60% protein with essential and non-essential amino acids (Bleakley and Hayes 2017). Pigments like astaxanthin, β-carotene, fucoxanthin, and others from algae like *Arthrospira, Haematococcus, Phaeodactylum*, and *Dunaliella* improve fish color and taste (Ahmad et al. 2023). While fish hatcheries use *Dunaliella* sp., *Isochrysis* sp., *Arthrospira* sp., and *Pavlova* sp. as larvae feed (Hodar et al. 2020), Nile tilapia (*Oreochromis niloticus*) is fed by *Nannochloropsis oculata* and *Schizochytrium* sp. to improve health (Souza et al. 2020). Another experiment substituted *Nannochloropsis* sp. and *Isochrysis* sp. for fish oil and fish meals in feed of rainbow trout (*Oncorhynchus mykiss*), a crucial model species for culturing salmon, *Isochrysis* sp. greatly raises the apparent digestibleness coefficients of undigested proteins, lipids, amino acids, and fatty acids (Sarker et al. 2020). White shrimp (*Fenneropenaeus indicus*) stomach harmful bacteria were effectively decreased by live* Tetraselmis suecica* (Regunathan and Wesley 2004). Many fish species can also have non-specific immune responses to infections caused by *Arthrospira* (Sheikhzadeh et al. 2019). The synthesis of blood cells, hemoglobin, albumin, and net protein is considerably increased in rainbow trout, which has served as a model for this (Yeganeh et al. 2015).

Humans, animals, and fish cannot synthesize polyunsaturated fatty acids PUFAs especially ω−3 and ω−6, so they must be consumed regularly and added as components for fish feed due to their inability to be made from saturated and unsaturated fatty acids (El‐Khodary et al. 2021). Fish feed-based algae enhance fish’s growth and immunity by increasing the ω−3/ω−6 ratio which improves the fatty acid profile (Chen et al. 2019). The important PUFAs in microalgal are arachidonic acid (ARA), eicosapentaenoic acid, n-3 (EPA), and docosahexaenoic acid, n-3 (DHA). For instance, *Thraustochytrium* sp. and *Schizochytrium* have around 22% DHA and 40% EPA, respectively, whereas *Phaeodactylum tricornutum* and *Nannochloropsis* sp. possess approximately 30% EPA and 39% DHA of total omega-3 fatty acids (Adarme-Vega et al. 2012). EPA is detected in* Nitzschia, Isochrysis,* and* Diacronema,* while ARA and DHA are found in* Cryptothecodinium*. When cultivated under stressful conditions, microalgae can produce significant quantities of lipids and possess a fatty acid composition that is good for nutrition.

On the other hand, for optimum growth, fish require meals with between 30 and 55% crude protein as well as specific amino acids (Wilson and Halver 1986). Hence, algae prove their efficiency once again as a food substitute for fish, as they contain protein concentrations fluctuating between 40 and 60 wt/wt % (Bleakley and Hayes 2017). One of the best sources of vegetarian protein is thought to be microalgae. It is well recognized that both essential and non-essential amino acids, which are both present in microalgae, have beneficial effects on health (Barka and Blecker 2016). Proline, arginine, glutamic acid, glycine, aspartic acid, cysteine, tyrosine, and serine are a few examples of non-essential amino acids.

The nutritional efficiency of microalgae also depends on their pigments, which are also used as colors in aquaculture. Astaxanthin and β-carotene are two examples of the carotenoids that* Arthrospira* and* Haematococcus* may produce (Sun et al. 2012), they have a potential effect on the quality and value of salmon and Asian tiger shrimp when added to feed. Large amounts of fucoxanthin are produced by *Phaeodactylum tricornutum*, which also gives gilthead seabream its golden color (Ribeiro et al. 2017), Although *Dunaliella saline* has significant concentrations of β-carotene, b-carotene, zeaxanthin, and chlorophylls a, b, and β-carotene, it is the most commonly produced species on a big scale, with as much as 15% DW (Paniagua-Michel 2015). Numerous studies have demonstrated that adding small amounts of *Arthrospira* can enhance the taste and color of a variety of fish species, including tilapia (Ahmad et al. 2023).

#### Effect of microalgae on the quality of aquaculture

The food industry devalues soft fillets, which is why fillet firmness is a key factor in the consumer acceptability of the aquaculture industry. Microalgae can reduce gaping in the fillet, when the connective tissue between muscle layers tears, it leaves the fillet with holes and a lack of stiffness. This condition is known as gapping. In Atlantic salmon, a 5% *Schizochytrium* sp. diet enhanced fillet superiority by falling gaping compared to a control diet (El-Sheekh et al. 2014; Kousoulaki et al. 2016). Moreover, studies have revealed that salmon fillets made with *Schizochytrium limacinum* have the same flavor and scent as salmon fillets made with common fish oil (Katerina et al. 2020). Mineral content in microalgae varies from 2.2 to 4.8% of their entire dry weight (Guedes et al. 2015). Gaping has been proven to be significantly reduced by inorganic minerals such as calcium, copper, iron, magnesium, potassium, phosphorus, sodium, sulfur, and zinc, as well as organic minerals like selenium and glutamate (Dineshbabu et al. 2019; Tavakoli et al. 2022). Microalgae are rich in minerals and were found to enhance the texture and flavor of salmon-fed diets (Guedes et al. 2015).

El-Sheekh et al. have revealed that *Arthrospira platensis* can be used to enhance the immunity ability of fish that consume it, as well as increase the growth rate and decrease the mortality rate in red tilapia group that fed diets that substituted 75% and 100% of fish meal (El-Sheekh et al. 2014). The survival rate was lower in the fish group fed a diet containing 0% dried *S. platensis*. On the other hand, histological examination showed the failure of completely replacing fishmeal with *S. platensis*, whereas myolysis and myophagia indicated muscle necrosis.

In conclusion, there needs to be more research on the effects of algae and algal compounds on fish qualitative parameters, such as gaping, flavor, etc. These parameters play a critical role in fish salability (Nagappan et al. 2021).

## Recommendation

Microalgae have emerged as a promising resource with diverse applications across multiple industries, aligning closely with several UN Sustainable Development Goals (SDGs). However, significant challenges and research needs must be addressed to fully realize their potential.

A primary concern in large-scale microalgae cultivation is the difficulty of maintaining continuous biomass production. This challenge is compounded by the risk of invasive species in large ponds and low light transmittance in concentrated cultures (Gao et al. 2019). Overcoming these obstacles is crucial for achieving SDG 2 (zero hunger) and SDG 12 (responsible consumption and production). To address these issues, future research should focus on isolating more promising algal strains and optimizing culture conditions for efficient pollutant elimination (Osman et al. 2023; Wang et al. 2024a). Genetic engineering may play a vital role in enhancing strain efficiency for contaminant removal, contributing to SDG 6 (clean water and sanitation) and SDG 14 (life below water). Recombinant DNA technology offers exciting possibilities for controlling the proportion of bioactive components in microalgae, potentially expanding their applications in the industrial, food, and medical sectors (Jagadevan et al. 2018). This aligns with SDG 3 (good health and well-being) and SDG 9 (industry, innovation, and infrastructure). However, to bring these innovations to a broader audience, strong collaborations between industry and public players are essential. These partnerships can drive innovation, streamline production processes, and expand microalgae use across various industries (Chew et al. 2017), supporting SDG 17 (partnerships for the goals).

The potential contribution of microalgae to the circular economy is an area that requires further investigation. Research in this direction could reveal new ways to integrate microalgae into sustainable production and consumption cycles, directly supporting SDG 12 (responsible consumption and production) and indirectly contributing to SDG 13 (climate action) (Venkata Mohan et al. 2019).

In the realm of renewable energy, microalgae show promise as a source of biofuels and hydrogen. Research efforts should focus on developing microalgae strains with increased photosynthetic capacity and improved activity of nitrogenase or hydrogenase enzymes, potentially leading to higher yields of hydrogen production (Show et al. 2017). This work directly supports SDG 7 (affordable and clean energy). However, before microalgae can be widely used as a biofuel feedstock, economically viable and cost-effective extraction techniques need to be developed and implemented.

To facilitate large-scale microalgae cultivation and the collection of valuable products like hydrogen and therapeutic bioactive compounds, the design and development of advanced biophotoreactors is crucial (Xue et al. 2013). This technological advancement could open up new avenues for industrial applications, contributing to SDG 9 (industry, innovation, and infrastructure).

Emerging research on the production and biodegradation of bioplastics from microalgae holds promise for addressing plastic pollution, aligning with SDG 14 (life below water) and SDG 15 (life on land) (Devadas et al. 2021). Additionally, innovative concepts like designing microalgae films to directly remediate air by releasing oxygen into the atmosphere, potentially replacing traditional factory chimneys, could significantly contribute to SDG 11 (sustainable cities and communities) and SDG 13 (climate action).

## Conclusion

Microalgae can directly or indirectly aid in achieving all SDGs and play a role in the circular economy. They are a sustainable, environmentally friendly energy source without competing with crops or requiring freshwater. Their rapid growth and low culture costs make them a potential nutrition source, contributing to reducing hunger and poverty (SDG 1 and 2). Microalgae produce carotenoids and natural compounds, enhancing health and well-being (SDG 3). They are commercially applied to provide sustainable energy like bioethanol, biohydrogen, biodiesel, and biogas, supporting clean energy access (SDG 7). Companies utilize microalgae for low-cost cosmetics manufacturing and biomedical products, promoting industry, innovation, and economic growth (SDG 8, 9, and 12). Microalgae can be grown using wastewater and atmospheric CO_2_, enabling wastewater treatment and CO_2_ mitigation, which is crucial for sustainable communities, preserving resources, and creating a sustainable environment (SDG 6, 11, 13, 14, and 15). Indirectly, microalgae support equal opportunities, education, and other SDGs (SDG 4–6, 10, and 14–17). In the circular economy, microalgae require only simple nutrients without complex substances or processes. Their biomass can generate clean water, bioactive substances, biofuels, and biofertilizers for agriculture, contributing to a sustainable cycle.

## Data Availability

Not applicable.
